# Quantum Dot Strategies Toward Performance Improvement of Perovskite Solar Cells

**DOI:** 10.3390/nano16150913

**Published:** 2026-07-24

**Authors:** Weixuan Liu, Chuangping Liu, Yu Ouyang, Qinghua Cao, Uliana Goga, Xiaoli Zhang, Smirnov Aliaksandr, Hui Liu

**Affiliations:** 1 School of Physics and Opto-Electronic Engineering, Guangdong Provincial Key Laboratory of Sensing Physics and System Integration Applications, Guangdong University of Technology, Guangzhou 510006, China; 2 Department of Micro- and Nanoelectronics, Belarusian State University of Informatics and Radioelectronics (BSUIR), 220013 Minsk, Belarus

**Keywords:** quantum dots strategies, carrier transport layers, active layers, UV conversion layers, perovskite solar cells

## Abstract

Perovskite solar cells (PSCs) have reached certified efficiencies exceeding 26%, yet the gap to the Shockley–Queisser limit and insufficient operational stability remain key obstacles to commercialization. Quantum dots (QDs) offer a versatile platform to address both challenges through their size-tunable bandgaps, high photoluminescence yields, and solution processability. This review systematically examines four QD integration strategies in PSCs: transport layer modification, active layer doping, UV conversion layers, and tandem sub-cells. The underlying mechanisms—including defect passivation, energy-level engineering, crystallization control, and ion migration suppression—are critically compared across these approaches. Despite significant advances, challenges persist, including the ligand–charge transport trade-off, the environmental toxicity of Pb/Cd-containing QDs, poor reproducibility, and the absence of standardized stability testing protocols. By providing a mechanism-oriented assessment across all device components, this review offers a clear framework for selecting appropriate QD strategies and identifies priority research directions. The perspective of QD strategies in this review provides a useful and significant reference for approaching the theoretical PCE limits of single-junction PSCs by reducing non-radiative recombination and improving light utilization, while QD-based tandem architectures offer a viable route toward surpassing the single-junction Shockley–Queisser limit.

## 1. Introduction

**Figure 1:**
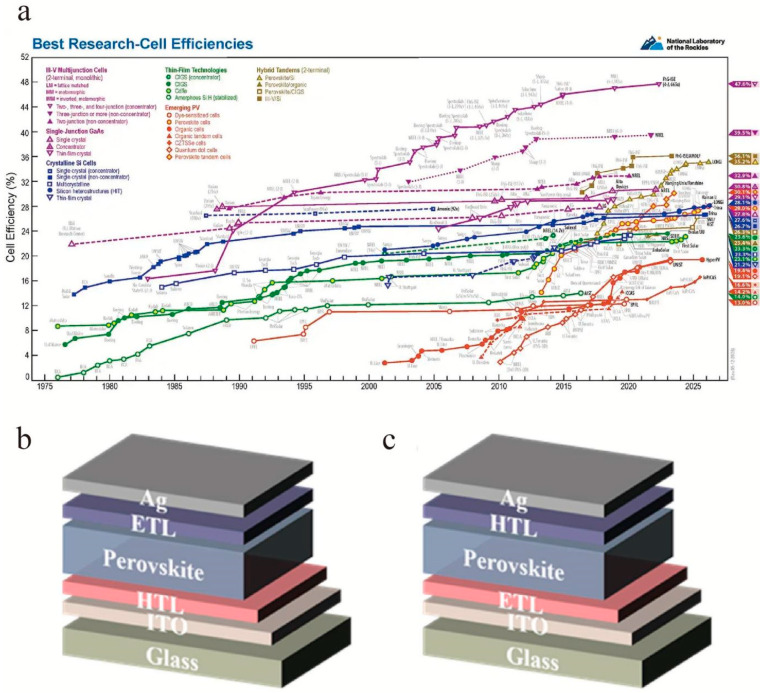
(**a**) Best research-cell efficiency chart in the world [1]; (**b**) planar p-i-n and (**c**) planar n-i-p structures of PSCs.

The global energy crisis has led to significantly increased research on clean energy sources such as solar energy. To maximize solar energy utilization, various types of solar cells have been developed, including silicon-based solar cells, organic solar cells, dye-sensitized solar cells, quantum dot solar cells, and more recently perovskite solar cells (PSCs) [1,2,3]. PSCs have attracted widespread attention and have advanced rapidly due to their excellent optoelectronic properties, simple fabrication, and remarkable achievements in power conversion efficiency (PCE) from 3.8% in 2009 to a record-certified 27.3% in 2026 (Figure 1a), making them one of the most popular clean renewable energy solutions [1]. Typically, PSCs consist of a perovskite active layer, carrier transport layers (CTLs) of electron transport layer (ETL) and hole transport layer (HTL), and electrodes. There are two main structures for PSCs: n-i-p and p-i-n. The p-i-n structure is Glass/ITO(FTO)/HTL/Perovskite/ETL/Ag (Au) (Figure 1b), and n-i-p is Glass/ITO(FTO)/ETL/Perovskite/HTL/Ag (Au) (Figure 1c). Common ETL materials include TiO_2_, SnO_2_, ZnO, C_60_ and its derivatives, and HTL materials constitute NiO*_x_*, PEDOT:PSS, PTAA, Spiro-OMeTAD, P3HT and so on. The active layer of organic–inorganic halide perovskites has the formula ABX_3_, where A typically is methylammonium (MA), formamidinium (FA), and/or cesium (Cs); B is lead (Pb) and/or tin (Sn); and X is iodine (I), bromine (Br), and/or chlorine (Cl).

However, the development of high-efficiency and long-lifetime devices is still severely limited by the existing drawbacks of perovskite films and device interlayers. Firstly, the perovskite layer, as the active layer of PSCs, primarily absorbs visible light and a small portion of near-infrared light [4]. The limited light absorption range constrains light utilization, resulting in insufficient charge carrier collection. Moreover, the existence of the Shockley–Queisser (SQ) efficiency limit prevents the efficiency of PSCs from increasing beyond 33% because of the inherent band structures and the corresponding energy loss of perovskites [5]. Furthermore, due to the degradation of PSCs under operating conditions and air atmosphere, their practical deployment requires simultaneous solutions to stability obstacles and further efficiency breakthroughs. In addition, PSCs are susceptible to degradation under operational conditions and ambient air exposure, necessitating concurrent advancements in both stability and efficiency for their practical deployment. To address the stability challenges in ambient air, one effective approach is the encapsulation of perovskite devices with protective materials. For instance, Raja et al. demonstrated that polymer-based encapsulation can effectively isolate perovskites from moisture and oxygen, substantially extending the device lifetime under ambient conditions [6]. In a related context, Konidakis et al. reviewed the development of advanced composite glasses, emphasizing that encapsulating perovskite nanocrystals within glass matrices offers a promising strategy to enhance their stability and mitigate lead toxicity, which is of particular relevance to the practical deployment of perovskite-based optoelectronic devices, including PSCs [7]. This glass-encapsulation strategy, though primarily demonstrated at the nanocrystal level, offers valuable insights for improving the long-term stability of full PSC devices. These encapsulation strategies, alongside other mitigation methods, are essential for bridging the gap between laboratory-scale achievements and real-world applications. Furthermore, the potential of QD-based strategies in reducing non-radiative recombination losses and improving device stability is analyzed. We also discuss how QD/perovskite tandem configurations may provide a pathway to surpass the single-junction SQ limit, while emphasizing that single-junction QD-modified PSCs alone cannot exceed this fundamental limit.

**Figure 2:**
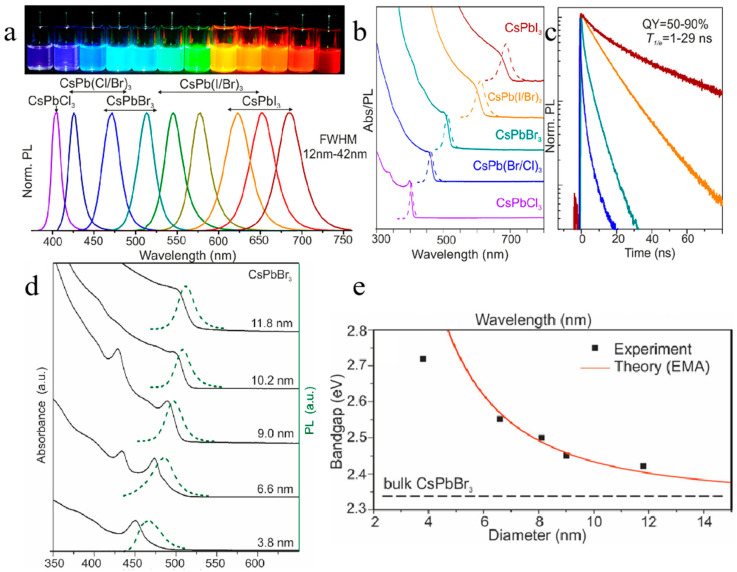
Light absorption dictated by composition and size of QDs. (**a**) Colloidal solutions in toluene under a UV lamp (λ = 365 nm) and representative PL spectra (λ_exc_ = 400 nm for all but 350 nm for CsPbCl_3_ samples). (**b**) Typical optical absorption and PL spectra. (**c**) Time-resolved PL decay curves of all samples except for the CsPbCl_3_ sample. (**d**) Quantum-size effects in the absorption and emission spectra of 5–12 nm CsPbBr_3_ NCs. (**e**) Experimental versus theoretical (effective mass approximation, EMA) size dependence of the band gap energy [8].

In this review, the term “quantum dots (QDs)” is primarily used to denote semiconductor nanocrystals that exhibit quantum confinement effects when their dimensions are smaller than the excitonic Bohr radius of the material. However, following common practice in the literature, some nanoscale materials (e.g., certain metal oxide nanoparticles) are also referred to as QDs when they are employed in analogous device architectures, even if strict quantum confinement is not explicitly demonstrated in the cited works. Where confinement is not demonstrable, the terms nanocrystals (NCs) and nanoparticles (NPs) are used interchangeably based on the original references. Quantum dots (QDs) have unique quantum confinement effects and excellent photovoltaic properties (e.g., high photoluminescence quantum efficiency, tunable wide bandgap, large absorption coefficient, and photostability, as shown in Figure 2), and have been widely applied in display technology, biological labeling, optoelectronic lighting, photonic computing, and solar cell devices [8,9,10,11,12,13]. The introduction of QDs into PSCs demonstrates numerous advantages that boost the optoelectronic performance of PSCs, including broadening light absorption, filling grain boundaries and passivating defects of perovskites, and facilitating carrier transport from perovskites to CTLs. To be more specific, the utilization of light in perovskite devices can be effectively improved by the broadened absorption range provided by the introduction of lower-bandgap QDs [14].

In addition, the introduction of QDs in PSCs enables surface modification of perovskite films through defect passivation and grain boundary filling [15]. The organic ligands on the surface of quantum dots offer numerous functional groups, which contribute to effective passivation of surface defects of perovskites and thus improve the quality of perovskite films and reduce energy loss caused by defects [16]. In terms of charge carrier transport, QDs can serve as buffer layers and as charge transport mediators to facilitate the energy-level alignment between the perovskite layer and CTLs, thereby accelerating the extraction and transport of charge carriers, stabilizing interfaces, suppressing interfacial recombination, and thus ultimately enhancing the performance of PSCs [17,18,19,20].

To gain deeper insights into these charge carrier dynamics and the underlying mechanisms within PSC devices, various ultrafast laser-based diagnostic techniques have been widely employed. These advanced characterization tools—including transient absorption spectroscopy (TAS), time-resolved photoluminescence (TRPL), and terahertz photoconductivity measurements—serve as figure-of-merit methods for evaluating and optimizing device performance. For instance, TAS was utilized to elucidate the long-range charge carrier diffusion lengths in organolead trihalide perovskites, providing critical guidance for thickness optimization in device design [21]. Wang et al. employed TRPL to probe the recombination dynamics and trap-state densities, revealing the impact of interfacial modification on carrier lifetimes [22]. More recently, Khan et al. applied advanced optical spectroscopic techniques to correlate charge carrier kinetics with device stability, offering valuable feedback for the rational design of stable PSCs [23]. These ultrafast techniques not only facilitate the understanding of fundamental photophysical processes but also provide actionable feedback for device engineering, thereby accelerating the development of PSCs with both high efficiency and long-term operational stability. Unlike previous reviews that focus on isolated aspects, this work provides a critical, mechanism-oriented comparison of QD strategies across all device components, clarifies the realistic potential and limitations—including the SQ limit for single-junction versus tandem devices—and identifies key bottlenecks to guide future research.

In this review, the crucial roles of the introduced QDs in PSCs are discussed, including their employment in ETL and HTL, application as a UV conversion layer, incorporation with the perovskite active layer as well as their application in constructing tandem solar cells. In addition, the potential of QD-based strategies in reducing non-radiative recombination losses and improving device stability is analyzed. We also discuss how QD/perovskite tandem configurations may provide a pathway to surpass the single-junction SQ limit, while emphasizing that single-junction QD-modified PSCs alone cannot exceed this fundamental limit. Finally, the conclusion and outlook for QD strategies toward PSCs are proposed, and key bottlenecks are identified to guide future research. For context, selected non-QD interfacial modifiers are briefly surveyed as performance benchmarks, but the central focus remains on QD-based strategies.

## 2. Quantum Dots Employed in CTLs

Carrier transport layers (CTLs) are essential components in the sandwich structure of PSCs, and they significantly influence and determine the efficiency of charge carrier extraction and transfer. However, problems such as interface defects, energy-level mismatch, and unsatisfactory carrier mobility for the CTLs largely impede the efficiency of PSCs. Therefore, selecting appropriate CTLs with good energy-level alignment and high carrier mobilities, as well as optimizing CTLs and interfaces through various strategies like passivation, is imperative for advancing the efficiency and stability of PSCs. The employment of QDs in CTLs or at the interfaces between perovskite absorbers and CTLs presents significant effects, including improving energy-level alignment, prolonging carrier lifetime, enhancing PL intensity, suppressing non-radiative recombination, and reducing charge transfer resistance at the interface [18,19,20]. This section focuses on QD-based strategies used in carrier transport layers and at CTL/perovskite interfaces. In this section, the term “QDs” is used only for nanoscale semiconductor materials for which quantum dot characteristics are explicitly reported in the original studies, such as PbS, CdSe, AgInS_2_, CuInS_2_, carbon-based QDs, MXene QDs, and perovskite QDs. Metal–oxide nanomaterials for which quantum confinement is not demonstrated are described as nanocrystals (NCs) or nanoparticles (NPs), following the terminology of the cited literature. Non-QD molecular or ionic modifiers are discussed only as comparative interfacial-engineering benchmarks and are explicitly labeled as non-QD materials.

### 2.1. QDs in ETLs

**Figure 3:**
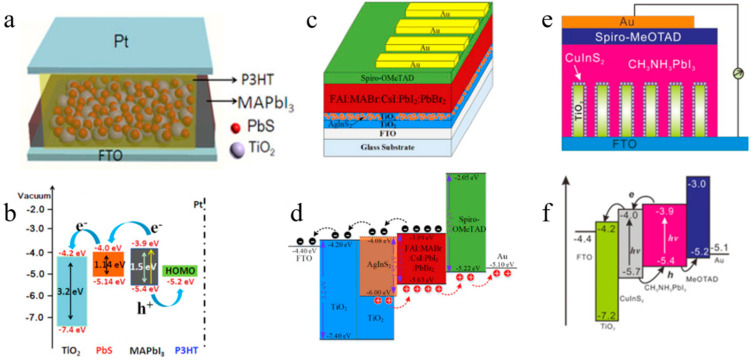
(**a**) Schematic of the hybrid perovskite solar cell based on TiO_2_/PbS/MAPbI_3_/P3HT/Pt, and (**b**) the energy level alignment in the hybrid perovskite solar cell [26]. (**c**) Schematic representation of perovskite solar cells with the device structure of FTO/TiO_2_-AgInS_2_-perovskite/Spiro-MeOTAD/Au. (**d**) Energy level alignment of the materials for the solar cells [27]. (**e**) Schematic representation of perovskite solar cells with the device structure of FTO/TiO_2_-CuInS_2_-perovskite/Spiro-MeOTAD/Au, and (**f**) energy level alignment of the materials for the solar cells [28].

The diverse QD-based and selected non-QD interfacial modifier strategies applied to ETLs can be broadly categorized by their primary working mechanisms: (i) interfacial defect passivation (e.g., C9, PBGH, STRS, FOA; non-QD modifiers), (ii) energy-level alignment and charge extraction enhancement (e.g., PbS, AgInS_2_, CuInS_2_, MXene QDs, SnO_2_ NCs/NPs, NbO_x_-SnO_2_ NCs, and Eu^3+^-doped SnO_2_), (iii) crystallization modulation and buried-interface engineering, and (iv) ion-migration suppression and stability enhancement. This classification provides a mechanistic framework for understanding how each material contributes to device performance. It should be noted that all core mechanistic discussions below center on QDs, and the listed non-QD passivators (C9, Eu^3+^, PBGH, STRS, FOA) are only briefly cited as comparative benchmarks. At the early stage, TiO_2_ is utilized as the ETL for n-i-p PSCs, but the large number of trap states in the dense TiO_2_ layer and its low conductivity severely limit the development of high-performance PSCs. Typically, defects at the interface can be passivated by introducing a thin organic layer such as [6,6]-phenyl-C_61_-butyric acid methyl ester (PCBM) between TiO_2_ and perovskite layers, which would reduce carrier recombination, improve perovskite film quality, and thus enhance the efficiency and stability of PSCs [24,25]. Apart from organic molecules, QD materials have been innovatively introduced to modify the TiO_2_ layer for higher efficiency and stability. Yang et al. introduced PbS QDs at the interface between TiO_2_ and perovskite layers (Figure 3a,b) [26]. The application of PbS QDs effectively reduces the damage to the CH_3_NH_3_PbI_3_ composition caused by the TiO_2_ layer, improves perovskite crystallization, enhances light absorption, facilitates electron transport, decreases electron recombination, and ultimately enhances device stability. The PbS QD-modified device retains 82% of its initial PCE after 97 h of storage in ambient air at room temperature (30–50% relative humidity) without encapsulation, whereas the control device without PbS QDs retains only 30% under the same conditions. Kaewprajak et al. employed a convective deposition technique to uniformly deposit AgInS_2_ QDs in TiO_2_ thin films, creating a dual ETL (TiO_2_/TiO_2_:AgInS_2_) for planar heterojunction PSCs (Figure 3c,d) [27]. This integration strategy effectively decreases defects at the interstices of TiO_2_ grains, enhances electron extraction and transfer from perovskite to TiO_2_, reduces hysteresis, and increases photocurrent. Gao et al. reported an interfacial modification strategy using CuInS_2_ QDs to enhance device performance (Figure 3e,f) [28]. CuInS_2_ QDs have a high absorption coefficient (~10^5^ cm^−1^), a proper bandgap (~1.6 eV), and low toxicity. The CuInS_2_ QDs are decorated on TiO_2_ nanorod arrays to obtain TiO_2_-CuInS_2_ nanorod arrays, which effectively enhance light absorption and improve the charge transfer process.

In recent years, with the improvement in the efficiency of PSCs, the TiO_2_ ETL has gradually been replaced by SnO_2_ colloidal dispersion, which requires a lower annealing temperature, has a larger bandgap and can create better energy-level alignment at the SnO_2_/perovskite interface, thus promoting photogenerated electron transfer from the perovskite layer to the SnO_2_ ETL [29,30,31]. However, the aqueous dispersion of SnO_2_ is not suitable for application in the inverted structure of PSCs due to the degradation of the perovskite layer caused by the water solvent, which would significantly lower the efficiency of the devices [19,32]. Therefore, SnO_2_ is widely used in the regular structure of PSCs and has achieved considerable progress.

**Figure 4:**
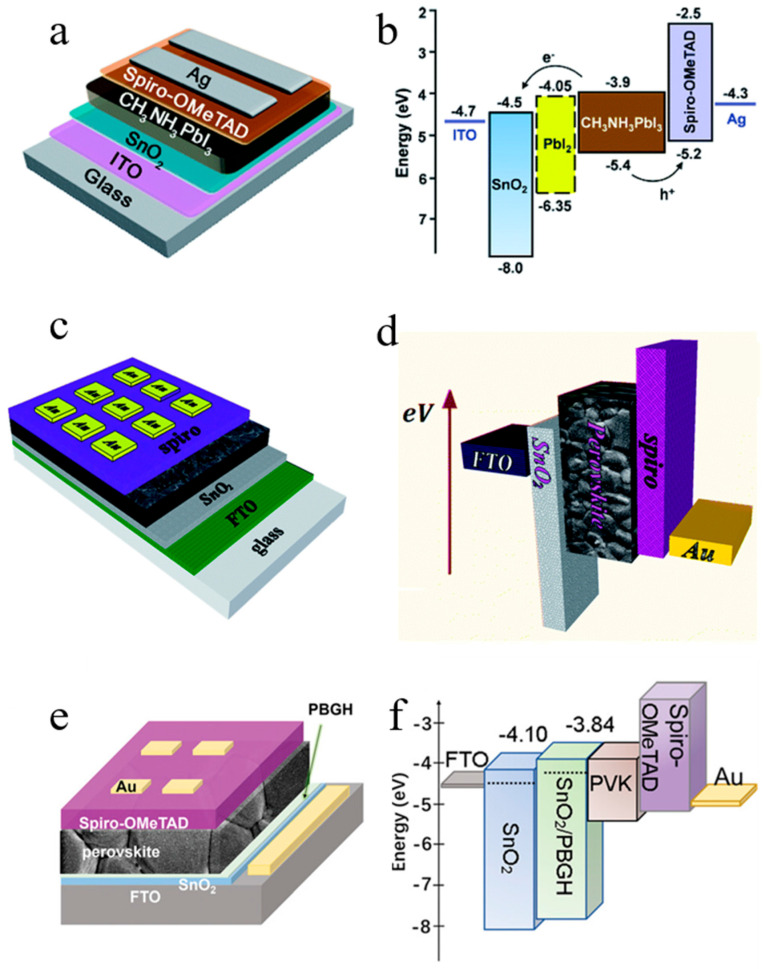
Device architecture (**a**) and energy diagram (**b**) of the ITO/SnO_2_/CH_3_NH_3_PbI_3_/spiro-OMeTAD/Ag cells. Reproduced with permission from Ref. [33]. (**c**) Device structure of the PSCs using Q-SnO_2_ and N-SnO_2_ as ETLs, and (**d**) the corresponding energy level alignment sketch of the PSCs [34]. (**e**) Schematic diagram of glass/FTO/SnO_2_/PBGH/FA_0.9_Cs_0.1_PbI_3_/Spiro-OMeTAD/Au PSC structure, and (**f**) the calculated energy band structure [35].

In 2015, Song et al. reported low-temperature solution-treated SnO_2_ as an ETL for planar PSCs (Figure 4a,b) [33]. The SnO_2_ layer is prepared by annealing at 150 °C, and a PCE of 13.0% is finally achieved for the PSCs. Liu et al. proposed a simple method of synthesizing SnO_2_ nanocrystal colloidal solution at room temperature by using an alcohol-based solvent and deionized water (Figure 4c,d) [34]. The NC colloidal solution is spin-coated and annealed to obtain an excellent uniform ETL, and results show that the SnO_2_ nanoparticle-based ETL enhances electron extraction and transfer, suppresses charge recombination, and finally helps achieve an average PCE of 18.6% for PSCs. However, several challenges still exist for SnO_2_ ETL applications (Figure 4e,f), such as the high concentration of defects at the interface between perovskite film and SnO_2_, leading to a poor buried interface and thus poor efficiency and stability of the device [35].

**Figure 5:**
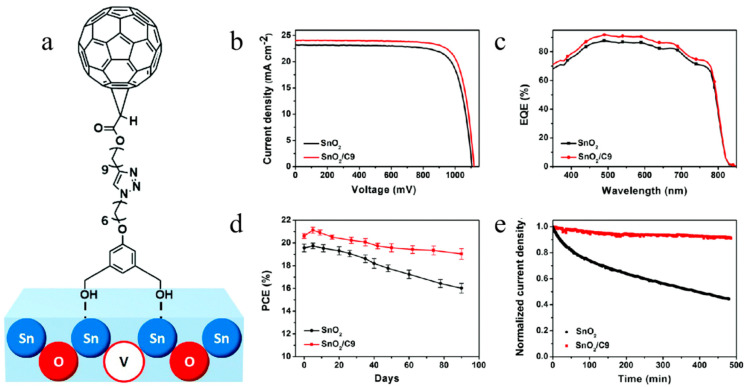
(**a**) Molecule structure of C9 and the schematic mechanism of oxygen vacancy-related defect passivation; (**b**) J-V curves of the best-performing device based on SnO_2_/C9 and the control device based on bare SnO_2_; (**c**) EQE spectra of devices; (**d**) ambient stability test of the un-encapsulated devices with and without the C9 modifying layer, stored under an ambient atmosphere; (**e**) photo and electric field stability test of the unencapsulated devices with and without the C9 modifying layer under continuous illuminated soaking in N_2_ atmosphere [36].

Substantial research has been conducted on SnO_2_-based PSCs over the past few years. As a comparative non-QD baseline, Liu et al. synthesized a fullerene derivative of C9 (non-QD) (as shown in Figure 5) to immobilize the interface of the SnO_2_ ETL in planar heterojunction PSCs [36]. C9 can effectively passivate the oxygen vacancy-related defects at the surface of SnO_2_ by forming Lewis adducts through the interaction of under-coordinated Sn in SnO_2_ and hydroxyl end-groups in C9, which successfully inhibits charge carrier recombination. Chen et al. proposed a method of doping Eu^3+^ in SnO_2_, which enhances electron mobility in the ETL, passivates trap defects at the grain boundaries, and finally leads to a high Voc of PSCs [37].

**Figure 6:**
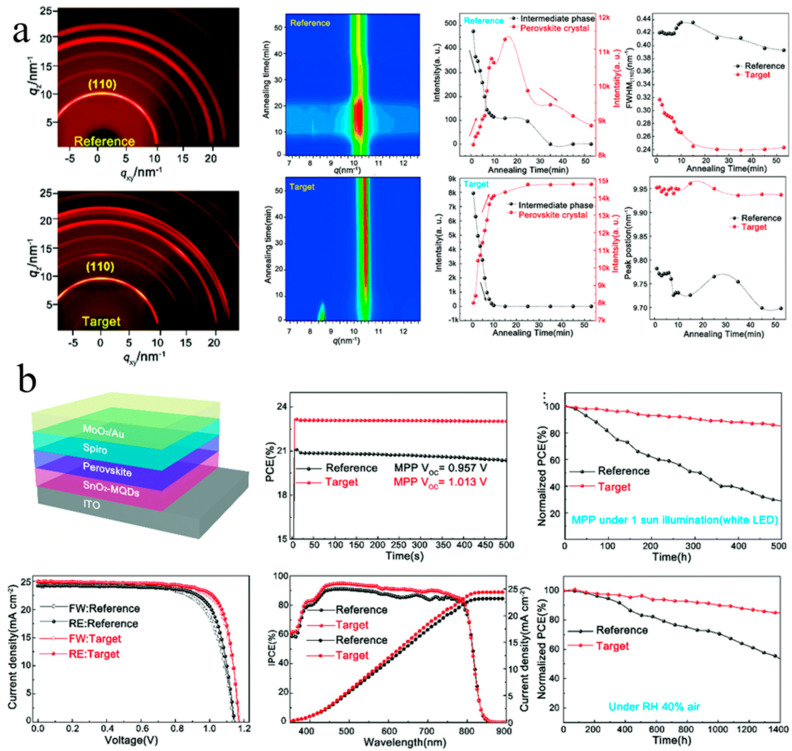
(**a**) GIXRD measurements of perovskite films, and (**b**) PV performance of the PSCs [38].

Yang et al. demonstrated that introducing MXene (Ti_3_C_2_T_x_) QDs modifies the interface of the SnO_2_ ETL and the perovskite layer [38]. The MXene QDs can effectively enhance charge carrier extraction and transfer and help form a high-quality and stable perovskite phase, finally leading to a steady-state PCE of up to 23.3% for the PSCs. As shown in Figure 6, the fundamental crystallization kinetics of perovskites formed on MQD-modified SnO_2_ (MQD-SnO_2_) reveal that the MQD-SnO_2_ ETL greatly increases the nucleation rate of perovskites during the initial spin-coating process and facilitates the formation of high-quality perovskite crystals, which ultimately contributes to the significantly improved PCE and stability of PSCs.

**Figure 7:**
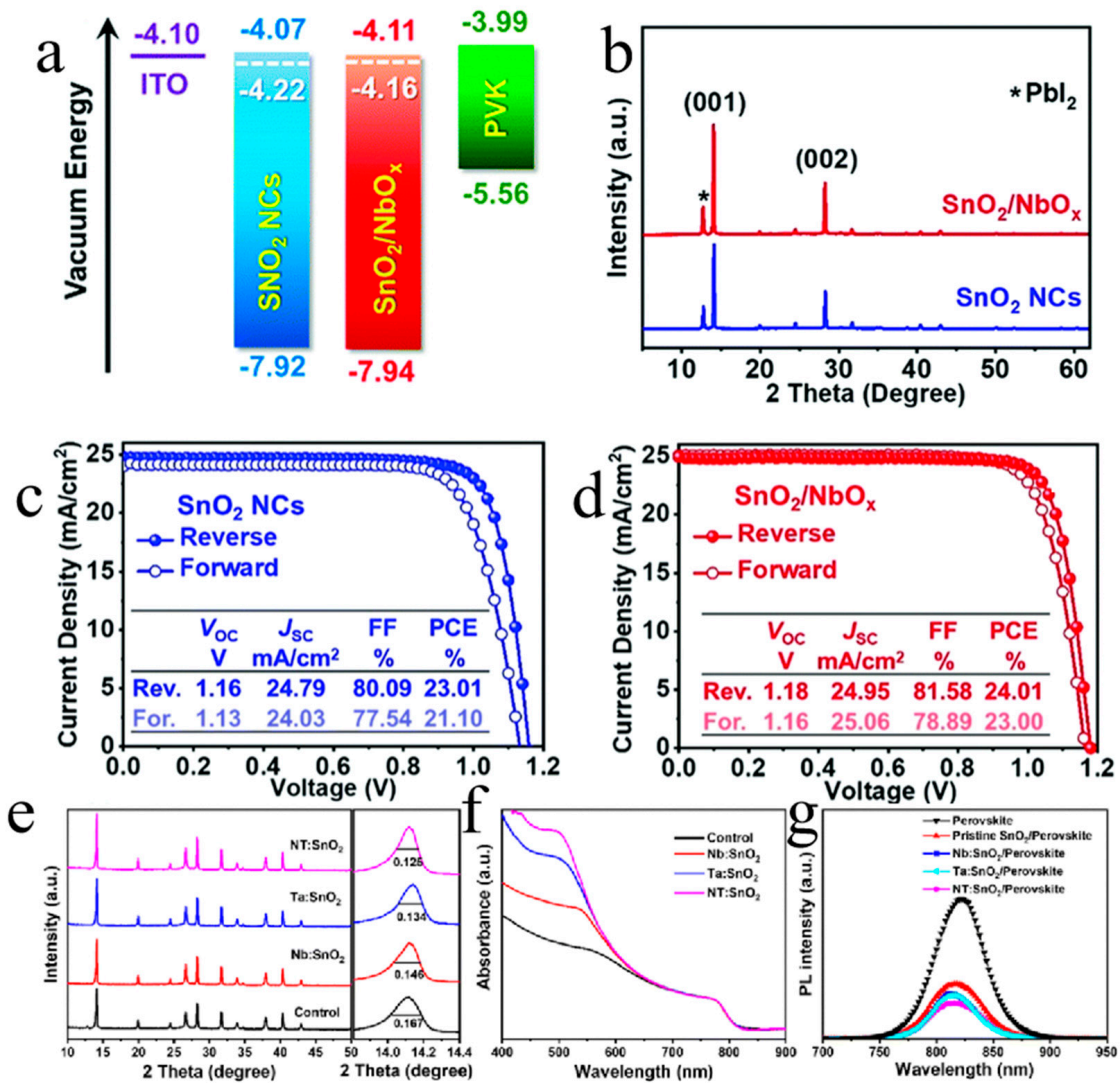
(**a**) Schematic energy band structures of SnO_2_ and SnO_2_/NbO*_x_* NCs in the devices, (**b**) XRD patterns of perovskite films on SnO_2_ and SnO_2_/NbO*_x_* ETMs, (**c**) SnO_2_ and (**d**) SnO_2_/NbO*_x_* ETMs under reverse and forward scans [39]. (**e**) XRD patterns, (**f**) UV-vis absorption spectra, and (**g**) PL spectra for perovskite films based on pristine and doped SnO_2_ [40].

In 2021, Yuan et al. developed an amorphous NbO_x_-encapsulated SnO_2_ nanocrystals (SnO_2_/NbO*_x_*) as the efficient ETL for PSCs and achieved a PCE of 24.01% (Figure 7a–d) [39]. Chen et al. effectively tuned the optoelectronic properties of SnO_2_ through an Nb^5+^ and Ta^5+^ co-doping strategy (denoted as NT:SnO_2_) [40]. The tuned SnO_2_ can modulate the subsequent crystallization of perovskites, leading to improved crystallinity and finally a PCE of 25.30% accompanied by a high fill factor of 84.51% (Figure 7e–g). Graphdiyne oxide can similarly affect the role of SnO_2_ in PSCs. Wang et al. added graphdiyne oxide (GDYO), nitrogen-doped GDYO (NGDYO), and fluorinated GDYO (FGDYO) into the SnO_2_ ETL [41]. They systematically explored the mechanism, and finally found that the strong interaction between doped SnO_2_ and PbI_2_ suppresses the crystallization of PbI_2_ in the perovskite layer, offering more chances for perovskite formation from the PbI_2_ precursor and leading to better crystallization of perovskites.

**Figure 8:**
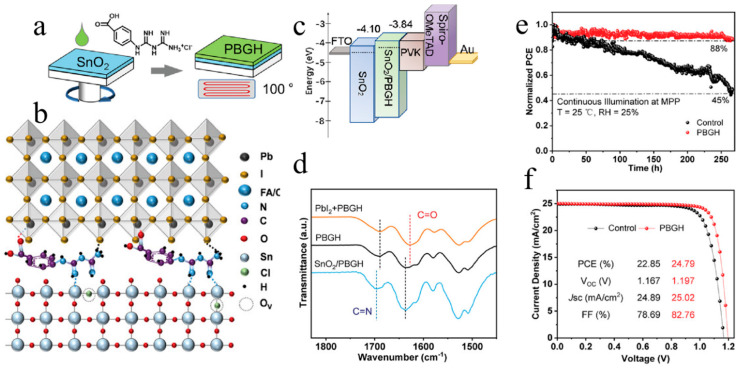
(**a**) Schematic diagram of the PBGH modification process. (**b**) Schematic diagram for the interaction mechanism of PBGH at the SnO_2_/perovskite interface. (**c**) The calculated energy band structure. (**d**) FTIR spectra of SnO_2_/PBGH, PBGH, and PbI_2_ + PBGH films. (**e**) Continuous MPP tracking for encapsulated control and PBGH-modified PSCs. (**f**) J-V curves [35].

In addition to inorganics, many organics are also used to modify the buried interface between SnO_2_ and perovskite. For example, Wu et al. used P-biguanylbenzoic acid hydrochloride (PBGH, non-QD) to modify the buried interface between SnO_2_ and perovskites [35]. As illustrated in Figure 8, PBGH can effectively passivate trap states of Sn dangling bonds and O vacancies at the surface of SnO_2_ through Lewis acid–base coordination, which effectively enhances the conductivity of SnO_2_ and accelerates electron extraction. Moreover, due to the strong interaction between PBGH and PbI_2_, it contributes to forming a high-quality perovskite film with low defect density.

**Figure 9:**
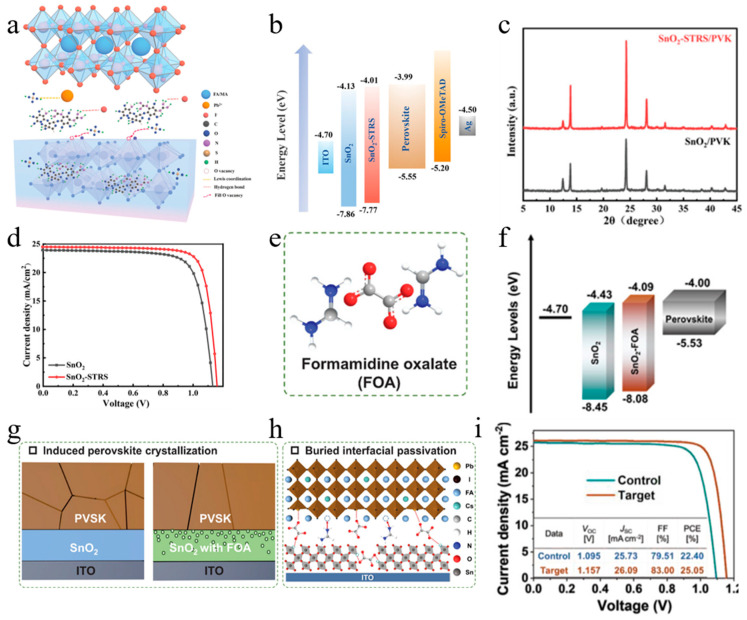
(**a**) Schematic diagram of the interaction of STRS with SnO_2_ and perovskite, (**b**) energy level diagram of perovskite solar cells, (**c**) XRD patterns of the perovskite film on SnO_2_ and SnO_2_-STRS substrates, and (**d**) J-V curves of the optimal SnO_2_ and SnO_2_-STRS PSCs [42]. (**e**) The 3D structure of FOA, (**f**) the schematic diagram of the energy level arrangement of the components used in the devices, (**g**) schematic illustration of the FOA distribution and its regulation for upper perovskite crystal growth, (**h**) the schematic diagram for the passivation function of FOA at the buried SnO_2_/perovskite interface, and (**i**) J-V curves of the PSCs based on pristine SnO_2_ and SnO_2_-FOA [43].

Yan et al. used an organic multifunctional aminoglycoside antibiotic to modify the buried interface of perovskite [42]. As presented in Figure 9a–d, they proposed to dope streptomycin sulfate (STRS, non-QD) molecules with multiple functional groups into the SnO_2_ ETL to inhibit the agglomeration of SnO_2_ nanoparticles, improve the electronic properties of SnO_2_, and reduce non-radiative recombination. Owing to the STRS, the interfacial residual tensile stresses are released and the interfacial energy levels are arranged in a more matched manner. Finally, STRS-SnO_2_-based PSCs achieve hysteresis-free, humidity- and thermal-stable performance. Ji et al. used formamidine oxalate (FOA, non-QD) to optimize the buried interface and regulate carrier dynamics of the device, leading to minimized defects, better interfacial contact and energy-level matching between perovskite and SnO_2_, and thus ultimately enhanced efficiency of PSCs (Figure 9e–i) [43].

**Figure 10:**
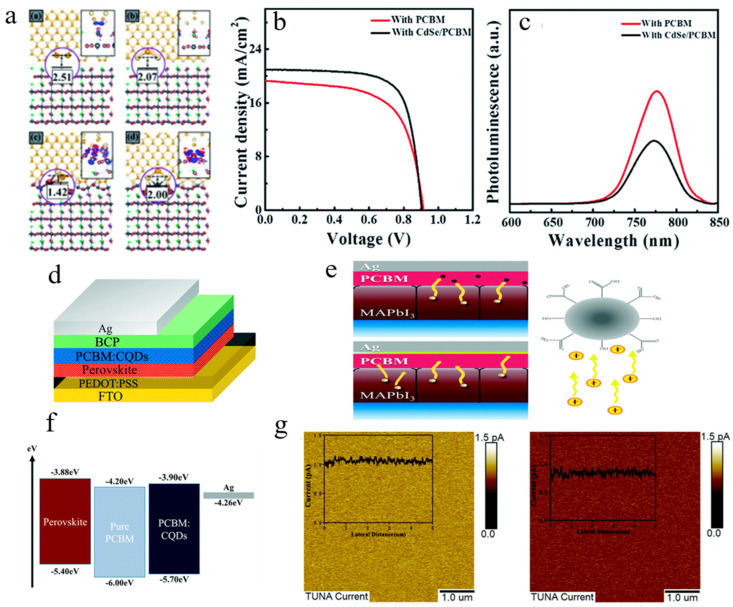
(**a**) DFT calculations between CdSe and perovskite, (**b**) J-V characteristics, and (**c**) PL spectra of the fabricated solar cells with PCBM and CdSe/PCBM as the ETL [44]. (**d**) Device architecture of the PHJ perovskite solar cell; (**e**) the proposed mechanism for preventing I^−^ from diffusing into a Ag electrode; (**f**) energy levels of CH_3_NH_3_PbI_3_, PCBM ETL and PCBM:CQD ETL; and (**g**) C-AFM images of ITO/PCBM:CQD and ITO/PCBM film with a scan size of 5 × 5 μm^2^, respectively [19].

Moreover, QDs such as CdSe and AgInS_2_ can also be used as additives in PCBM. Zeng et al. utilized CdSe QDs to form CdSe/PCBM composites as the ETL for CH_3_NH_3_PbI_3x_Cl_x_ PSCs (Figure 10a–c) [44]. The application of the CdSe/PCBM ETL effectively reduces the roughness of the perovskites formed on it, leading to a dense and smooth perovskite film. In addition, DFT modeling demonstrates that the hybridization of atomic orbitals between CdSe QDs and perovskites significantly enhances the structural stability of perovskites. Zhu et al. used carbon quantum dots (CQDs) doped into PCBM as the optimized ETL for inverted PSCs (Figure 10d–g) [19]. They showed that the conductivity, the electron mobility, and the charge extraction ability are largely improved by the quasi-spherical CQD doping, and that CQD doping helps to prevent I^−^ from undergoing interfacial diffusion, which effectively improves the operational stability of PSCs. Specifically, the unencapsulated devices with PCBM:GQD ETL maintain over 80% of their initial PCE after 300 h of continuous AM 1.5G full-spectrum illumination (including the UV component), whereas the reference devices without GQDs drop to below 50% under identical conditions.

The side-by-side comparison between CdSe and CQDs/GQDs in these two studies presents a compelling case not only in device performance but also from an environmental standpoint. CdSe, as a cadmium-based compound, introduces the same toxicity and regulatory concerns discussed earlier for Cd-containing QDs. Although the quantity of QDs used in ETL modification is relatively small, the potential release of Cd^2+^ ions during device fabrication, operation, or after disposal remains a non-negligible risk, particularly given that perovskite solar cells are already under intense scrutiny for their intrinsic lead content. The additional introduction of cadmium could further complicate the already challenging end-of-life management and may hinder commercialization under tightening regulations such as the EU RoHS Directive. In stark contrast, the CQDs/GQDs employed by Zhu et al. are composed entirely of carbon, a non-toxic and earth-abundant element, thereby circumventing heavy-metal-related regulatory barriers. Moreover, the superior operational stability achieved by CQDs/GQD-doped devices—maintaining over 80% of initial PCE after 300 h of full-spectrum illumination versus below 50% for the reference—suggests that the “green” option does not necessarily compromise device longevity; rather, it may offer dual benefits in both environmental compatibility and practical durability. This observation reinforces the notion that low-toxicity alternatives, when properly engineered, can rival or even surpass their toxic counterparts in critical performance metrics, making them increasingly attractive for future PSC development.

However, the intrinsic toxicity of Pb and Cd poses serious environmental, health, and safety (EHS) challenges and has triggered stringent regulatory restrictions at both international and regional levels. For instance, the exemptions for cadmium-based quantum dots in display applications under the EU RoHS (Restriction of Hazardous Substances) Directive have been significantly tightened, with expiration scheduled between 2025 and 2027. This renders the commercialization pathways for Pb/Cd-containing quantum dots in photovoltaic products subject to fundamental compliance risks. Furthermore, potential leakage of these toxic elements throughout the device lifecycle, the environmental fate of degradation byproducts, and the lack of mature end-of-life recycling solutions constitute practical barriers to technological deployment. Therefore, in discussing the optoelectronic performance of these toxic-element-containing quantum dots, it is imperative to systematically assess their environmental fate, regulatory constraints, and commercialization risks, while simultaneously reviewing the recent advances and performance bottlenecks of low-toxicity alternatives, such as carbon-based quantum dots, CuInS_2_, InP, and other related systems. Compared with other QD schemes, ETL QDs mainly optimize interfacial charge extraction without regulating perovskite bulk crystallization. The key parameters of representative quantum dot materials employed as electron transport layers or interfacial modifiers in PSCs are summarized in Table 1.

It should be noted that the quantitative comparison in Table 1 is most feasible for ETL modifications, where reported device parameters are relatively uniform in terms of device architecture and testing conditions. For active-layer doping, UV management, and tandem configurations, the reported studies vary more widely in perovskite compositions, fabrication protocols, and characterization conditions, making direct cross-comparison in a single table less meaningful.

### 2.2. QDs in HTLs

**Figure 11:**
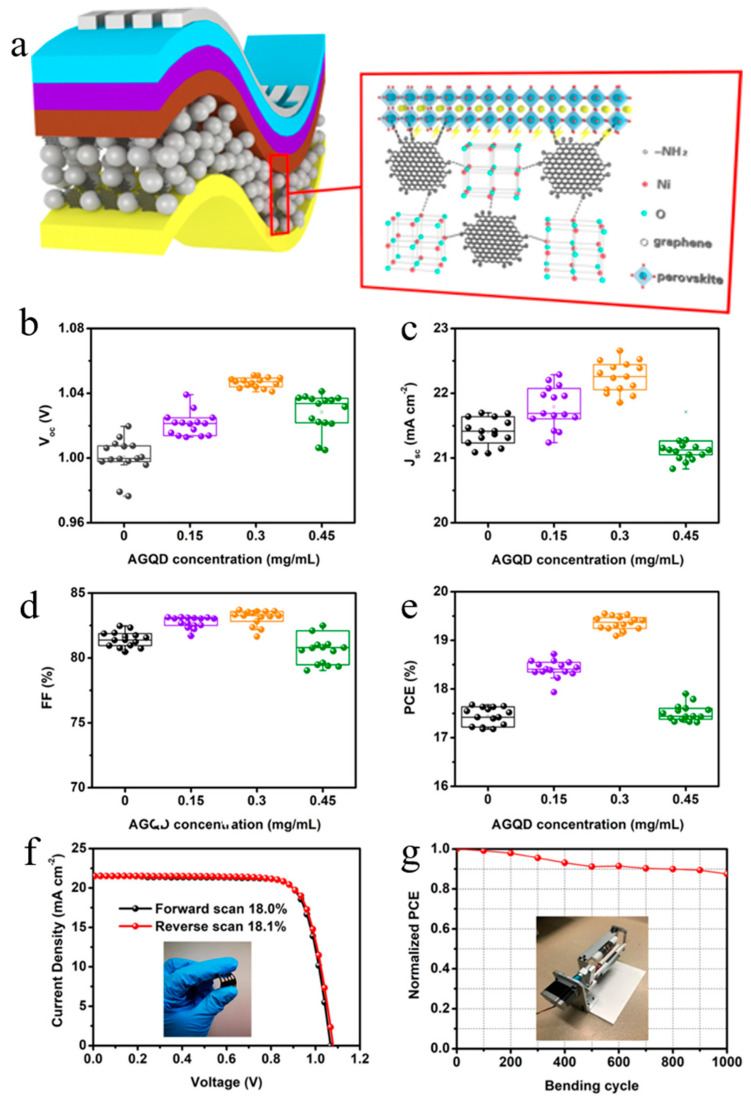
(**a**) Schematic illustration of the interaction of GQDs with NiO_x_ and perovskite. (**b**) V_OC_, (**c**) J_SC_, (**d**) fill factor (FF), and (**e**) PCE of perovskite solar cells fabricated with different concentrations of AGQDs in the NiO_x_ precursor ink. (**f**) J-V curves of the best flexible PSC. The inset shows the photograph of the cell. (**g**) Evolution of the normalized PCE of the A-NiO_x_-based flexible PSC as a function of bending cycle [45].

The QD-based strategies in HTLs can be grouped into several mechanistic categories: (i) conductivity enhancement (e.g., AGQDs in NiO_x_, MWCNT:NiO in spiro-OMeTAD), (ii) hydrophobic protection and humidity stability (e.g., MWCNT:NiO, perovskite QD interlayers), (iii) improved hole extraction (e.g., perovskite QDs forming cascade energy levels with undoped o-HTLs), and (iv) suppression of ion migration and interfacial defect passivation (e.g., MWCNT:NiO limiting Li^+^/FA^+^/MA^+^ loss, NiO_x_/SAMs composite systems). This categorization highlights the distinct roles that QDs and QD-assisted interfacial layers play in optimizing HTL performance. QD materials are not only applicable to the modification of ETLs but also play a significant role in HTLs. Wang et al. developed a novel strategy of using amino-functionalized graphene quantum dots (AGQDs) as additives in NiO_x_ HTLs for highly efficient flexible PSCs (Figure 11) [45]. Three types of QD additives, including imidazole-functionalized graphene QDs, hydroxyl-functionalized graphene QDs and AGQDs, were studied, and the addition of AGQDs to NiO_x_ yielded the best improvement in PSC performance, which is attributed to the high electrical conductivity, superior chemical stability, improved band structure of AGQD-optimized NiO_x_ HTL, as well as the enhanced quality of the perovskite film.

**Figure 12:**
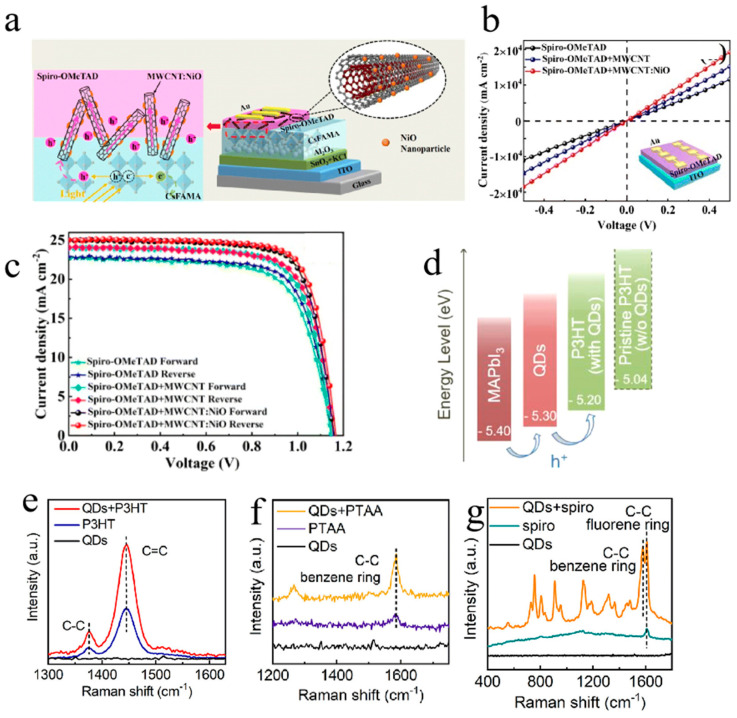
(**a**) Device architecture and the schematic illustration of charge transfer and transport at the CsFAMA/spiro-OMeTAD + MWCNT:NiO layer. (**b**) J-V curves. (**c**) J-V curves of the best devices of PSCs based on different HTLs [20]. (**d**) Schematic of energy band alignment. (**e**–**g**) Optimization of dopant-free o-HTLs on QD interlayers [46].

The application of spiro-OMeTAD HTL in PSCs typically suffers from poor ambient conductivity, unfavorable ion migration, and numerous defects at the perovskite/HTL interface. Rong et al. successfully designed NiO_x_ nanoparticles-modified multi-walled carbon nanotubes (MWCNT:NiO) as p-type doping additives in Li-TFSI/tBP-based spiro-OMeTAD HTL for efficient and stable PSCs (Figure 12a–c) [20]. Benefiting from the high conductivity and hydrophobicity of MWCNTs and the interaction between NiO nanoparticles and spiro-OMeTAD/Li-TFSI molecules, the moisture resistance of MWCNT:NiO modified spiro-OMeTAD HTL is significantly enhanced. The unencapsulated PSCs with MWCNT:NiO maintain 91% of their initial efficiency after 1200 h of aging in ambient air at room temperature (30–50% relative humidity), while the control devices lose about 62% of their initial PCE under the same conditions. Moreover, the MWCNT:NiO additive not only enhances hole extraction/transfer by improving interfacial energy-level alignment and passivating perovskite defects, but also effectively limits Li^+^ migration and decreases the FA^+^/MA^+^ loss, thus hindering the degradation of perovskites. Cheng et al. successfully increased the hole mobility of commercially available organic hole transport layer (o-HTL) materials without ion doping while maintaining the stability of PSCs (Figure 12d–g) [46]. They demonstrated that incorporating perovskite QDs between perovskites and undoped o-HTLs (P3HT, PTAA, and Spiro-OMeTAD) can significantly enhance the performance of PSCs, outperforming the effect of lithium doping. The strategy of introducing QDs plays multiple roles, including passivating the perovskite surface by eliminating trap states, facilitating hole extraction from perovskite to undoped o-HTLs through the formation of cascade energy levels, improving the hole mobility of the undoped o-HTLs by modulating the orientation of polymers, and considerably enhancing the thermal/moisture/light stability of undoped o-HTLs.

For comparative purposes, we also briefly survey recent progress in self-assembled molecules (SAMs, non-QD) as HTLs, as these represent a complementary approach to interface engineering. Many widely used self-assembling molecules have been employed to modify NiO_x_ HTLs, including 2PACz, Br-2PACz, Me-4PACz, Meo-2PACz, Meo-4PADBC, ODPA, TBT-DBA, and Me-PACz+PC, among others. Table 2 summarizes the key performance parameters of representative SAM-modified NiO_x_ HTLs as a reference for benchmarking against QD-based approaches. Lin et al. investigated the modification of the NiO_x_ surface by 2PACz and ethanolamine [47]. The 2PACz has excellent hole selectivity, and the ethanolamine molecule can passivate the perovskite and cover the NiO_x_ surface. The dual surface modification finally results in a significant enhancement of device stability. Zhu et al. synthesized a new SAM of MeO-4PADBC, which is immobilized on the NiO_x_ layer of PSCs [48]. The interfacial structure immobilizes SAM molecules at the NiO_x_/perovskite interface and generates a thermally stable HTL for PSCs, which results in a high V_OC_ of 1.19 V and a desired PCE of 25.6%. Yan et al. modified the buried interface of NiO_x_-based PSCs by doping Me-4PACz with phosphorylcholine chloride (PC) to form Co-SAM to improve the coverage of monolayers [49]. Phosphoric acid groups and chlorine ions (Cl^−^) in PC can inhibit the surface defects of NiO_x_, while quaternary ammonium ions and Cl^−^ can fill the organic cation and halogen vacancies and defects at the bottom of the perovskite film. Thus, Co-SAM successfully promotes the growth of perovskite crystals, inhibits non-radiative recombination, accelerates carrier transport, and reduces the residual stress of perovskites.

## 3. QDs as UV Conversion Layer

The effectiveness of a UV conversion layer relies on several key photophysical criteria. First, the material should exhibit a high photoluminescence quantum yield (PLQY) to maximize photon conversion efficiency. Second, strong spectral overlap between the layer’s absorption and the UV region (typically 300–400 nm) is required, while its emission should overlap well with the perovskite absorption window to ensure efficient utilization of down-converted photons. A sufficiently large Stokes shift is desirable to minimize reabsorption losses. Third, the layer must maintain high optical transparency in the visible range to avoid parasitic absorption. Fourth, adequate photochemical stability under prolonged UV exposure and compatibility with device encapsulation are essential for practical applications. It is also important to distinguish between three related but distinct mechanisms: down-conversion (one high-energy photon is converted into two or more lower-energy photons, with a quantum yield > 100%), luminescent down-shifting (one high-energy photon is absorbed and re-emitted as one lower-energy photon, with a quantum yield ≤ 100%), and UV filtering (absorption of UV light without re-emission, functioning as a protective optical filter). These distinctions have important implications for device design and performance expectations and should be carefully considered when selecting QD materials for UV management in PSCs. The organic components of perovskite films suffer from degradation when exposed to high-energy UV photons for a long time, and various strategies have been developed to alleviate this challenge. Adding an additional energy-down-shift (EDS) layer that converts ultraviolet light to visible light beneath the perovskite layer has been widely shown to effectively reduce damage to perovskites from ultraviolet light. Various luminescent materials (e.g., QDs, rare-earth ions/compounds, and organic dyes) have been utilized for EDS applications to increase UV stability as well as device efficiency.

**Figure 13:**
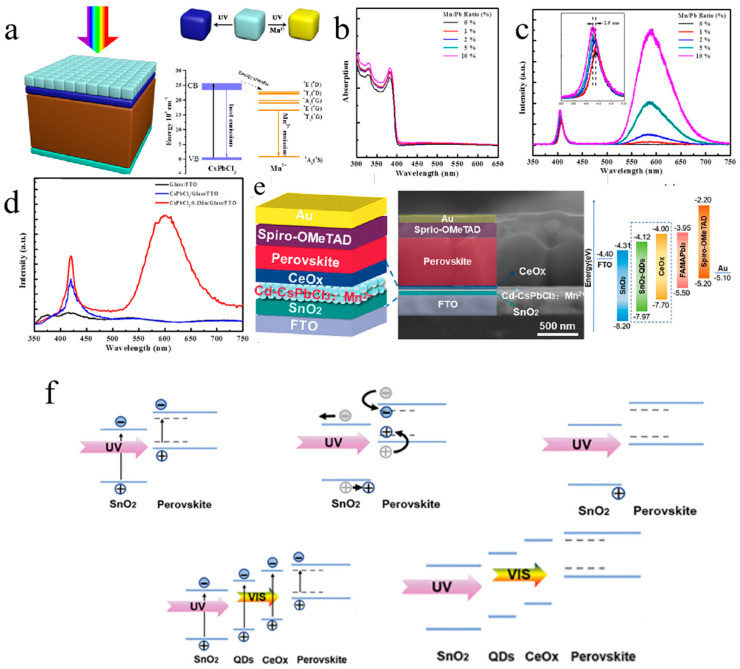
(**a**) Schematic structure of solar cells coated with a CsPbCl_3_:0.1Mn layer, (**b**) absorption spectra, (**c**) emission spectra (excited by 365 nm) of CsPbCl_3_:*x*Mn QD solution with different Mn^2+^ concentrations, and (**d**) emission spectra (excited by 365 nm) of glass/FTO, CsPbCl_3_/glass/FTO, and CsPbCl_3_:0.1Mn/glass/FTO [58]. (**e**) The completed device structure of the PSC, the corresponding cross-sectional SEM image of the completed PSC, and the energy level diagram of the PSCs. (**f**) Schematic diagram of the degradation mechanism of PSCs by UV light [59].

Wang et al. synthesized CsPbCl_3_:Mn QDs with high a quantum yield (~60%) and significant Stokes shift (>200 nm) and employed them as EDS layers in PSCs. The CsPbCl_3_:Mn QDs can effectively convert UV light (300–400 nm) into visible light at around 590 nm, which successfully increases the PCE and stability of PSCs (Figure 13a–d) [58]. However, the application of the EDS layer faces a problem of mutual diffusion between the EDS layer and the perovskite layer, seriously limiting the performance advancement of PSCs. Liu et al. introduced a dual-ETL approach that sandwiches the Cd-CsPbCl_3_:Mn^2+^ QDs within the ETL gap (Figure 13e,f) [59]. This strategy not only reduces the interfacial energy-level shift and prevents the diffusion of QDs into the perovskite layer, but also improves the nucleation and crystallization of perovskites, which leads to improved Voc, FF, and significantly improved photostability. QDs as EDS layers can also be applied in silicon solar cells. To address the low utilization of near-ultraviolet light, Song et al. mixed Mn^2+^/Er^3+^-doped CsPbCl_3_ QDs (CsPbCl_3_:Mn^2+^, Er^3+^ QDs) with ethylene-(vinyl acetate) to form an EDS layer for silicon solar cells (Figure 13e,f) [60]. Nitrogen-doped graphene QDs are also effective in converting harmful UV light into visible photons, thereby increasing the efficiency of solar cells [61,62,63,64,65,66,67].

As summarized in Table 3, the stability tests vary significantly in terms of testing conditions, duration, and encapsulation status. While all three strategies demonstrate improved stability compared to their respective control devices, the lack of standardized testing protocols makes it difficult to directly compare the effectiveness of different QD strategies. Nevertheless, these results confirm that QD incorporation can effectively enhance device stability under ambient storage and light illumination conditions, highlighting the potential of QD-based strategies for practical applications of PSCs.

## 4. QDs Employed in Active Layer

### 4.1. QD Doping in Perovskites

Recently, QDs have been doped as additives in perovskite precursors. The small size of QDs enables them to be doped into the grain boundaries of perovskites, while the abundant organic ligands on the surface of QDs can interact with perovskites to passivate defects at the grain boundaries, reducing carrier loss during transport [62,63]. Moreover, incorporating a small number of QDs substantially enhances the crystallinity of perovskites by providing extra nucleation sites, thereby enhancing the photovoltaic performance of PSCs [64,65,66,67]. There are different ways to introduce QDs into perovskites, such as directly dissolving them in a precursor, adding them in an antisolvent, or post-treating them on the surface of perovskite films [68,70,71,72,73].

**Figure 14:**
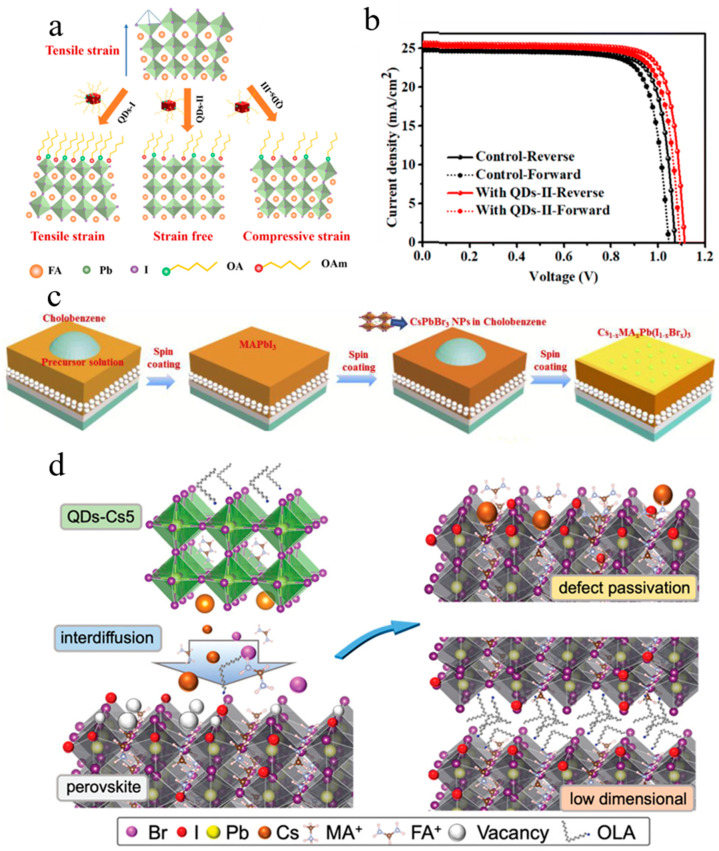
(**a**) Schematic representation of the strain state of the control film and the QD-I-, QD-II-, and QD-III-treated films. (**b**) Forward and reverse scans of the control and QD-II-treated PSCs [74]. (**c**) Schematic fabrication of perovskite films incorporated with CsPbBr_3_ NPs [68]. (**d**) Schematic the of proposed ionic defect passivation mechanism achieved through QD-C modification [77].

Han et al. developed a one-step method to prepare MAPbI_3_-PbS QD hybrid films by homogeneously dispersing prefabricated PbS QDs in the MAPbI_3_ precursor before growing them on mesoporous TiO_2_ substrates [14]. With the hybridization of QDs, the final perovskite film shows higher quality with larger grain size, enhanced crystallinity, improved morphology, and slightly longer absorption range, as well as a significantly increased carrier mobility, which largely facilitates charge transfer and collection. Xu et al. applied colloidal CdSe QDs as additives to form high-quality CsPbI_2_Br films under ambient conditions (Figure 14a,b) [74]. The addition of CdSe QDs effectively contributes to promoting nucleation and forming sufficient homogeneous nuclei for the crystallization process of perovskites. Benefiting from the high-quality CsPbI_2_Br perovskite films with the application of QDs, the champion PCE of the optimized PSC device increased from 12.73% (reference) to 14.49%. An effective strategy was introduced by dispersing inorganic QDs in the antisolvent of chlorobenzene to form perovskites, which led to high-quality films with reduced grain boundaries and trap states [75]. The antisolvent passivates the surface trap states by promoting rapid crystallization, leading to an increase in grain size, as the quantum dots can fill all the pinholes [76]. Gao et al. proposed a novel strategy by introducing CsPbBr_3_ nanoparticles (NPs) into chlorobenzene antisolvent to improve the quality of MAPbI_3_ thin films in terms of film morphology and crystallinity (Figure 14c) [68]. The CsPbBr_3_ NPs act as nucleation centers for the growth of perovskites and form a passivation layer of Cs_1-*y*_MA*_y_*PbI_3-*x*_Br*_x_* on top of the perovskites, which effectively reduces charge recombination and improves charge transfer. Xie et al. reported an ion-defect passivation strategy using multi-cation hybridized halide perovskite QDs and pointed out that ionic defects on the film surface and grain boundaries of perovskites can be effectively eliminated through a process of ionic solid-state interdiffusion (Figure 14d) [77]. QDs also have the ability to adjust the energy band structure of perovskites, resulting in enhanced carrier transport from perovskites to carrier transport layers. An integrated structure was studied based on CsPbI_2_Br and CsPbI_3_ QDs with a gradient bandgap formed by proper band-edge bending, which facilitates charge carrier transfer and helps achieve an efficient bilayer thin-film device without interlayer parallel connection [71]. Yang et al. developed a passivation strategy to improve the surface quality of perovskite films by combining triple cationic perovskites with perovskite QDs during the film formation process [70]. Due to the similar crystalline structure of perovskite QDs and perovskite films, the lattice distortions of different components are prevented, resulting in a decrease in surface defects. Meanwhile, the morphology, surface electronic properties, and crystallinity of perovskites are greatly improved, which in turn effectively enhances carrier dynamics [15,16,17,31,68,69,73,74,77,78,79,80,81,82,83,84].

Unlike CTL QDs that only tune device interfaces, active-layer QDs simultaneously adjust perovskite grain quality and light absorption, accompanied by unique ligand-induced aggregation risks that are absent in thin-layer QD modification. While QD doping exhibits significant advantages in crystallization modulation and defect passivation, this strategy also entails several nontrivial challenges. First, the dispersibility of QDs in the perovskite precursor or antisolvent directly affects doping uniformity; aggregation may lead to local compositional inhomogeneity and the formation of additional trap states. Second, the long-chain organic ligands commonly present on QD surfaces (e.g., oleic acid, oleylamine) ensure colloidal stability, but their insulating nature may introduce charge transport barriers at grain boundaries, offsetting the charge extraction benefits that QDs are expected to provide. Short-chain ligands or inorganic ligands can improve electronic coupling but often sacrifice colloidal stability and process compatibility—a trade-off that lies at the core of optimizing QD doping strategies. Furthermore, lattice mismatch and interdiffusion between QDs and perovskites may induce phase instability and interfacial strain, compromising long-term operational stability. Reproducibility also remains a non-negligible engineering challenge, as batch-to-batch variations in QD synthesis and the narrow processing window for doping procedures may limit practical application. Therefore, the incorporation of QDs into the active layer is inherently a double-edged sword—the ultimate outcome depends critically on the delicate regulation of QD size, surface chemistry, doping concentration, and processing conditions.

### 4.2. QDs as Active Layer in Tandem Solar Cells

The application of tandem structures is an effective approach to exceeding the SQ limit. Due to their excellent photovoltaic properties, QDs are considered a promising candidate as the active materials of a sub-cell in tandem solar cells, which extend the absorption range and make it possible for the PCE of solar cells to exceed the SQ limit. Lead sulfide (PbS) QDs show a highly tunable bandgap and strong absorption in the infrared region, making them suitable candidates for constructing optimal QDs/perovskites tandem solar cells. Zhang et al. first reported stable and efficient tandem solar cells by combining MAPbI_3_ perovskites with PbS QDs and finally achieved perfectly stacked tandem cells by carefully adjusting the thicknesses of the sub-cells [84]. Although the introduction of a PbS QD sub-cell enhances infrared absorption and effectively eliminates the hysteresis effect of perovskites, the performance of the tandem solar cell is still lower than that of the single-junction cell. To improve the efficiency of tandem solar cells, Li et al. employed magnetron-sputtered MS ITO front electrodes and reactive-plasma-deposited RPD ITO rear electrodes to construct semi-transparent perovskite (ST-PVSK) solar cells. The ST-PVSK/QD 4T tandem solar cells finally achieved the highest efficiency of 26.12%, and both ST-PVSK and QD solar cells show excellent stability [69].

However, the efficient operation of perovskite/QD tandem solar cells relies on the synergistic optimization of several key factors. Current matching is a core requirement for two-terminal (2T) tandem structures, where the overall current is limited by the sub-cell with the lower current output. This necessitates precise regulation of light absorption distribution between the wide-bandgap perovskite top cell and the narrow-bandgap PbS QD bottom cell. Optical losses primarily arise from parasitic absorption in transparent electrodes, reflection losses at various functional layers, and optical absorption in the intermediate recombination layers, which can be mitigated through anti-reflection coatings and grating structures. Regarding transparent electrodes, magnetron-sputtered ITO and reactive plasma deposition (RPD) ITO have been employed to balance high near-infrared transmittance and conductivity, while aluminum-doped zinc oxide (AZO) has been introduced to enhance carrier extraction from the PbS QD sub-cell. The recombination layer (also referred to as the tunnel junction or intermediate connector) must simultaneously provide a low-resistance electrical connection, high optical transparency, and adequate solvent barrier capability to prevent damage to the bottom cell during top-cell fabrication [85]. In terms of bandgap selection, the combination of a conventional perovskite (~1.55 eV) with ~0.95 eV PbS QDs extends the absorption spectrum to approximately 1300 nm. For charge extraction, the energy levels of PbS QDs contract with increasing dot size, requiring matching electron transport layers (e.g., magnetron-sputtered ZnO) and hole transport layers to ensure efficient carrier collection. Finally, the operational stability of PbS QDs themselves remains a challenge, with surface oxidation and ligand desorption being the primary degradation mechanisms [86]. Strategies such as CdS shell coating have been explored to enhance stability, although they may compromise charge transfer efficiency.

To provide a more comprehensive assessment of the relative advantages and limitations of perovskite/QD tandems, it is instructive to briefly compare them with other mainstream tandem technologies. Perovskite/silicon tandem solar cells benefit from the mature industrial chain and excellent stability of crystalline silicon bottom cells, making them the tandem architecture closest to commercialization in the near term. Recent work by Er-Raji et al. demonstrated that optimizing the self-assembly of hole-selective monolayers enables fully textured perovskite/silicon tandem cells to approach 30% efficiency [87]. Flexible perovskite/silicon tandem devices have also achieved a certified efficiency of 33.4% on a 1 cm^2^ area [88]. All-perovskite tandem solar cells have recently surpassed the 30% efficiency milestone, with Lin et al. reporting a PCE of 30.6% (certified steady-state 30.1%) through a dipolar passivation strategy that suppresses non-radiative recombination at the buried interface of the narrow-bandgap sub-cell and extends the carrier diffusion length to 6.2 μm [89]. This marks the first time that polycrystalline thin-film solar cells have exceeded the 30% efficiency threshold. By contrast, perovskite/QD tandem solar cells utilize all-inorganic PbS QDs as the narrow-bandgap bottom cell, offering inherent advantages in stability. Moreover, the bandgap of QDs can be continuously tuned by size control, providing greater flexibility for spectral matching. However, this approach currently lags behind the other two routes in efficiency (26.12% for the 4T configuration), and the uniform deposition of QDs over large areas remains challenging. Quantitatively, 4T QD tandem devices reach higher efficiency than all single-junction QD-modified PSCs, yet they face exclusive current-matching bottlenecks not found in single-junction QD strategies. In summary, perovskite/QD tandems possess unique advantages in stability and bandgap tunability, but still require further improvements in efficiency and process maturity.

## 5. Conclusions and Outlook

This review is focused on quantum dot modification strategies for perovskite solar cells. Non-QD interface passivators and SAMs are included as comparative benchmarks to establish performance baselines and to highlight the specific advantages of QD-based approaches; they are not treated as independent research subjects. Quantum dots (QDs), with their unique quantum confinement effect and attractive photovoltaic properties, have emerged as a versatile class of materials for enhancing PSC performance. This review summarizes QD-based strategies across four integration routes: CTL modification, active layer doping, UV conversion layers, and tandem sub-cells. Among these, QD modification of transport layers—particularly carbon-based QDs in PCBM ETLs and MXene QDs in SnO_2_ ETLs—stands closest to practical application due to simple fabrication and consistent efficiency gains. Defect passivation and energy-level alignment are most consistently correlated with PCE improvements across all configurations. For stability, carbon-based QDs in ETLs (>80% retention after 300 h) and MWCNT:NiO in HTLs (91% retention after 1200 h) provide the most meaningful gains. PbS and CdSe QDs offer superior performance but raise serious toxicity and regulatory concerns under EU RoHS, while carbon-based and I–III–VI QDs offer safer alternatives at the cost of lower efficiencies. The wide variation in stability testing protocols (ambient vs. N_2_, encapsulated vs. unencapsulated) makes cross-study comparisons unreliable, calling for adoption of ISOS standards.

Looking forward, the most urgent research directions are as follows: (i) developing lead-free QDs that match PbS/CdSe performance; (ii) resolving the ligand–charge transport trade-off through ligand engineering; (iii) establishing reproducible synthesis and large-area deposition protocols; and (iv) integrating environmental safety into early-stage material design. Addressing these challenges will be essential for QD-based strategies to fulfill their potential as a transformative platform for next-generation PSCs. Therefore, the perspective of QD strategies toward PSCs in this review is useful and significant for approaching the theoretical PCE limits of single-junction PSCs. Moreover, the integration of QDs into tandem solar cell architectures presents a promising strategy to surpass the single-junction Shockley–Queisser limit, offering a realistic pathway toward next-generation high-efficiency photovoltaics.

In light of the above discussions, we firmly believe that the incorporation of quantum dots into future perovskite solar cells represents a highly worthwhile research direction that merits sustained investment from both academic and industrial sectors. This conviction is grounded in several compelling considerations. From a fundamental perspective, QDs offer a unique combination of functionalities—including defect passivation, spectral down-shifting, energy-level engineering, and crystallization modulation—that cannot be simultaneously achieved by conventional additives. This multifunctionality addresses multiple performance-limiting factors within a single material system, providing a cost-effective and operationally simple pathway for device enhancement.

From a practical standpoint, the solution-processable nature of QDs makes them readily compatible with existing thin-film fabrication infrastructure, thereby lowering the barrier for industrial adoption. Moreover, the rapidly expanding library of QD compositions (e.g., lead-free perovskite QDs, carbon-based QDs, and I–III–VI group QDs) offers ample opportunities for tailoring material properties to meet specific device requirements, including environmental sustainability and long-term operational stability.

Critically, the integration of QDs into tandem solar cell architectures opens a realistic avenue to surpass the Shockley–Queisser limit of single-junction devices—a goal that is fundamentally unattainable by standalone perovskite absorbers. As such, QD-based strategies are not merely incremental improvements but represent a transformative approach that could redefine the performance ceiling of photovoltaic technologies.

Therefore, we conclude that investing research efforts in QD-modified PSCs is not only justified but essential for the continued advancement of next-generation photovoltaics. We encourage future studies to prioritize the resolution of remaining challenges—particularly concerning long-term stability, large-area fabrication, and environmental safety—so as to fully unlock the immense potential of this promising combination.

## Figures and Tables

**Table 1:** Summary of QD-based and selected non-QD interfacial modifiers applied in electron-transport layers of perovskite solar cells.

Material/Classification	ETL Base	Device Architecture	PCE (%)	Stability Improvement	Ref.
PbS QDs	TiO_2_	n-i-p	9.5	Prolonged stability	[26]
AgInS_2_ QDs	TiO_2_	n-i-p (planar)	13.1	Improved device stability	[27]
CuInS_2_ QDs	TiO_2_ nanorod arrays	n-i-p	18.6	Enhanced stability and charge extraction	[28]
SnO_2_ NCs/NPs	SnO_2_	n-i-p (planar)	18.6 (avg.)	Improved uniformity	[34]
Fullerene derivative (C9)	non-QD fullerene derivativeSnO_2_	n-i-p (planar)	21.3	Good device stability	[36]
Eu^3+^-doped SnO_2_	non-QD doped oxide SnO_2_	n-i-p	20.14	High Voc	[37]
MXene (Ti_3_C_2_T_x_) QDs	SnO_2_	n-i-p	23.3 (steady-state)	Enhanced stability	[38]
NbO_x_-encapsulated SnO_2_ NCs	non-QD oxide NCs SnO_2_	n-i-p	24.01	Remarkable stability	[39]
Nb^5+^/Ta^5+^ co-doped SnO_2_	non-QD doped oxide SnO_2_	n-i-p	25.30	Outstanding device stability	[40]
GDYO/NGDYO/FGDYO	non-QD graphdiyne modifiers SnO_2_	n-i-p	21.23	Enhanced interfacial	[41]
PBGH	non-QD molecular modifier SnO_2_	n-i-p	24.79	Improved high-temperature/humidity and light stability	[35]
STRS	non-QD molecular modifier SnO_2_	n-i-p	22.89	Humidity/thermal stable	[42]
CdSe QDs	PCBM	Inverted (p-i-n)	15.1	Improved charge extraction	[44]
Carbon quantum dots (CQDs)	PCBM	Inverted (p-i-n)	18.1	Improved stability	[19]

**Table 2:** Summary of PSC performance based on NiO_x_ modified with SAMs (champion device).

HTL	PCE (%)	Jsc (mA/cm^2^)	Voc (V)	FF (%)	Ref.
NiO*x*/MeO-4PADBC	25.6	25.4	1.19	84.6	[50]
NiO*x*/Me-4PACz+PC	25.1	26.0	1.18	82.5	[49]
NiO*x*:Cu/MeO-2PACz	23.5	20.6	1.70	71	[51]
NiO*x*/ODPA	18.8	22.8	1.09	75.6	[52]
NiO*x*/TBT-BA	24.8	24.9	1.19	83.7	[53]
NiO*x*/Trp	23.8	25.3	1.14	82.3	[54]
NiO*x*/amine-2PACz	22.0	23.2	1.19	80.2	[47]
NiO*x*/Me-4PACz	17.3	22.6	1.04	73.1	[55]
NiO*x*/NCS	25.1	24.8	1.17	86.2	[56]
NiO*x*/I-2PACz	11.1	9.2	1.51	80.6	[57]

Abbreviations: NCS, 1,3-dimethyl-benzoimidazol-2-thione); ODPA, octadecylphosphonic acid; I-2PACz, (2-(3,6-diiodo-9H-carbazol-9-yl)ethyl)phosphonic acid. Me-4PACz+PC denotes Me-4PACz doped with PC.

**Table 3:** Summary of stability outcomes for QD-modified PSCs.

QD Strategy	Stability Type	Test Conditions	Duration	PCE Retention	Ref.
PbS QDs at TiO_2_/perovskite interface	Ambient storage	Ambient air, room temp (30–50% RH), unencapsulated	97 h	~82% (vs. 30% for control)	[26]
MWCNT:NiO in spiro-OMeTAD	Ambient storage	Ambient air, room temp (30–50% RH), unencapsulated	1200 h	91% (vs. 38% for control)	[20]
GQDs in PCBM	Light stability (full-spectrum)	AM 1.5G, full-spectrum including UV, unencapsulated	300 h	>80% (vs. <50% for control)	[19]
Active layer: CsPbBr_3_ NPs in MAPbI_3_	Ambient storage	Ambient air, RT, unencapsulated	1000 h	~90%	[68]
UV layer: CsPbCl_3_:Mn QDs as EDS	UV stability	UV irradiation, 5 mW/cm^2^	100 h	85%→97%	[58]
Tandem: Perovskite/PbS QD 4T	Storage stability	Inert conditions	2220 h	PbS: no degradation; PVSK: ~95% retention	[69]

## Data Availability

No new data were created or analyzed in this study. Data sharing is not applicable to this article.

## References

[1] Best Research-Cell Efficiency Chart|Photovoltaic Research|NLR. https://www.nlr.gov/pv/cell-efficiency.

[2] Luo W., Wen H., Guo Y., Yin T., Tan H., Zhang Z., Si S., Zhang Z., Wu H., Huang S. (2024). Simultaneous Ultraviolet Conversion and Defect Passivation Stabilize Efficient and Operational Durable Perovskite Solar Cells. Adv. Funct. Mater..

[3] Tang H., Shen Z., Shen Y., Yan G., Wang Y., Han Q., Han L. (2024). Reinforcing self-assembly of hole transport molecules for stable inverted perovskite solar cells. Science.

[4] Ji R., Zhang Z., Deconinck M., Hofstetter Y.J., Shi J., Paulus F., Raval P., Reddy G.N.M., Vaynzof Y. (2024). Spontaneous Formation of 1D/3D Perovskite Heterojunctions for Efficient Inverted Perovskite Solar Cells. Adv. Energy Mater..

[5] Guo Z., Jena A.K., Kim G.M., Miyasaka T. (2022). The high open-circuit voltage of perovskite solar cells: A review. Energy Environ. Sci..

[6] Raja S.N., Bekenstein Y., Koc M.A., Fischer S., Zhang D., Lin L., Ritchie R.O., Yang P., Alivisatos A.P. (2016). Encapsulation of perovskite nanocrystals into macroscale polymer matrices: Enhanced stability and polarization. ACS Appl. Mater. Interfaces.

[7] Konidakis I., Karagiannaki A., Stratakis E. (2022). Advanced composite glasses with metallic, perovskite, and two-dimensional nanocrystals for optoelectronic and photonic applications. Nanoscale.

[8] Protesescu L., Yakunin S., Bodnarchuk M.I., Krieg F., Caputo R., Hendon C.H., Yang R.X., Walsh A., Kovalenko M.V. (2015). Nanocrystals of Cesium Lead Halide Perovskites (CsPbX_3_, X = Cl, Br, and I): Novel Optoelectronic Materials Showing Bright Emission with Wide Color Gamut. Nano Lett..

[9] Park S., Song S., Kim S.S., Song J. (2021). Charge Modulation Layer and Wide-Color Tunability in a QD-LED with Multiemission Layers. Small.

[10] Bang S.Y., Fan X., Jung S., Yang J., Shin D.-W., Suh Y., Lee T., Lee S., Choi H.W., Occhipinti L. (2020). Highly Stable and Scalable Blue QD-LED via an Evaporated TiO_2_ Thin Film as an Electron Transport Layer. Adv. Opt. Mater..

[11] Dong R., Bi C., Dong Q., Guo F., Yuan Y., Fang Y., Xiao Z., Huang J. (2014). An Ultraviolet-to-NIR Broad Spectral Nanocomposite Photodetector with Gain. Adv. Opt. Mater..

[12] Qin H., Meng R., Wang N., Peng X. (2017). Photoluminescence Intermittency and Photo-Bleaching of Single Colloidal Quantum Dot. Adv. Mater..

[13] Zhang G., Yuan C., Li X., Yang L., Yang W., Fang R., Sun Y., Sheng J., Dong F. (2022). The mechanisms of interfacial charge transfer and photocatalysis reaction over Cs_3_Bi_2_Cl_9_ QD/(BiO)_2_CO_3_ heterojunction. Chem. Eng. J..

[14] Han J., Luo S., Yin X., Zhou Y., Nan H., Li J., Li X., Oron D., Shen H., Lin H. (2018). Hybrid PbS Quantum-Dot-in-Perovskite for High-Efficiency Perovskite Solar Cell. Small.

[15] Zhang S., Guo R., Zeng H., Zhao Y., Liu X., You S., Li M., Luo L., Lira-Cantu M., Li L. (2022). Improved performance and stability of perovskite solar modules by interface modulating with graphene oxide crosslinked CsPbBr_3_ quantum dots. Energy Environ. Sci..

[16] Yang X., Zhao W., Li M., Ye L., Guo P., Xu Y., Guo H., Yu H., Ye Q., Wang H. (2022). Grain-Boundaries-Engineering via Laser Manufactured La-Doped BaSnO_3_ Nanocrystals with Tailored Surface States Enabling Perovskite Solar Cells with Efficiency of 23.74%. Adv. Funct. Mater..

[17] Wang H., Song Y., Dang S., Jiang N., Feng J., Tian W., Dong Q. (2020). Reducing Photovoltage Loss in Inverted Perovskite Solar Cells by Quantum Dots Alloying Modification at Cathode Contact. Sol. RRL.

[18] Kim J.M., Lee B.S., Hwang S.W. (2020). High-Performance Core/Shell of ZnO/TiO_2_ Nanowire with AgCl-Doped CdSe Quantum Dots Arrays as Electron Transport Layer for Perovskite Solar Cells. Molecules.

[19] Zhu X., Sun J., Yuan S., Li N., Qiu Z., Jia J., Liu Y., Dong J., Lv P., Cao B. (2019). Efficient and stable planar perovskite solar cells with carbon quantum dots-doped PCBM electron transport layer. New J. Chem..

[20] Rong Y., Jin M., Du Q., Shen Z., Feng Y., Wang M., Li F., Liu R., Li H., Chen C. (2022). Simultaneous ambient long-term conductivity promotion, interfacial modification, ion migration inhibition and anti-deliquescence by MWCNT:NiO in spiro-OMeTAD for perovskite solar cells. J. Mater. Chem. A.

[21] Wehrenfennig C., Eperon G.E., Johnston M.B., Snaith H.J., Herz L.M. (2014). High charge carrier mobilities and lifetimes in organolead trihalide perovskites. Adv. Mater..

[22] Wang L., McCleese C., Kovalsky A., Zhao Y., Burda C. (2014). Femtosecond time-resolved transient absorption spectroscopy of CH_3_ NH_3_ PbI_3_ perovskite films: Evidence for passivation effect of PbI_2_. J. Am. Chem. Soc..

[23] Khan M.T., Almohammedi A., Kazim S., Ahmad S., Grätzel M., Ahmad S., Kazim S. (2021). Charge carrier dynamics in perovskite solar cells. Perovskite Solar Cells.

[24] Li H., Shi W., Huang W., Yao E.-P., Han J., Chen Z., Liu S., Shen Y., Wang M., Yang Y. (2017). Carbon Quantum Dots/TiO*_x_* Electron Transport Layer Boosts Efficiency of Planar Heterojunction Perovskite Solar Cells to 19%. Nano Lett..

[25] Zhang X., Guan Y., Zhang Y., Yu W., Wu C., Han J., Zhang Y., Chen C., Zheng S., Xiao L. (2022). Achieving Small Temperature Coefficients in Carbon-Based Perovskite Solar Cells by Enhancing Electron Extraction. Adv. Opt. Mater..

[26] Yang Y., Wang W. (2015). Effects of incorporating PbS quantum dots in perovskite solar cells based on CH_3_NH_3_PbI_3_. J. Power Sources.

[27] Kaewprajak A., Kumnorkaew P., Sagawa T. (2019). Improved photovoltaic performance and device stability of planar heterojunction perovskite solar cells using TiO_2_ and TiO_2_ mixed with AgInS_2_ quantum dots as dual electron transport layers. Org. Electron..

[28] Gao F., Dai H., Pan H., Chen Y., Wang J., Chen Z. (2018). Performance enhancement of perovskite solar cells by employing TiO_2_ nanorod arrays decorated with CuInS_2_ quantum dots. J. Colloid Interface Sci..

[29] Yang G., Chen C., Yao F., Chen Z., Zhang Q., Zheng X., Ma J., Lei H., Qin P., Xiong L. (2018). Effective Carrier-Concentration Tuning of SnO_2_ Quantum Dot Electron-Selective Layers for High-Performance Planar Perovskite Solar Cells. Adv. Mater..

[30] Wang Z., Shi C., Wang Z., Xiao L., Wu T., Yu X., Ma L., Chen X., Zhang J., Lei H. (2022). Solution-processed Fe_2_-xMgxO_3_ ternary oxides for interface passivation in efficient perovskite solar cells. Chem. Eng. J..

[31] Guo X., Lu C., Zhang W., Yuan H., Yang H., Liu A., Cui Z., Li W., Hu Y., Li X. (2024). In Situ Surface Sulfidation of CsPbI_3_ for Inverted Perovskite Solar Cells. ACS Energy Lett..

[32] Chang J., Feng E., Feng X., Li H., Ding Y., Long C., Lu S., Zhu H., Deng W., Shi J. (2024). Bridging buried interface enable 24.67%-efficiency doctor-bladed perovskite solar cells in ambient condition. Nano Res..

[33] Song J., Zheng E., Bian J., Wang X.-F., Tian W., Sanehira Y., Miyasaka T. (2015). Low-temperature SnO_2_ -based electron selective contact for efficient and stable perovskite solar cells. J. Mater. Chem. A.

[34] Liu H., Chen Z., Wang H., Ye F., Ma J., Zheng X., Gui P., Xiong L., Wen J., Fang G. (2019). A facile room temperature solution synthesis of SnO_2_ quantum dots for perovskite solar cells. J. Mater. Chem. A.

[35] Wu M., Duan Y., Yang L., You P., Li Z., Wang J., Zhou H., Yang S., Xu D., Zou H. (2023). Multifunctional Small Molecule as Buried Interface Passivator for Efficient Planar Perovskite Solar Cells. Adv. Funct. Mater..

[36] Liu K., Chen S., Wu J., Zhang H., Qin M., Lu X., Tu Y., Meng Q., Zhan X. (2018). Fullerene derivative anchored SnO_2_ for high-performance perovskite solar cells. Energy Environ. Sci..

[37] Chen Y., Zuo X., He Y., Qian F., Zuo S., Zhang Y., Liang L., Chen Z., Zhao K., Liu Z. (2021). Dual Passivation of Perovskite and SnO_2_ for High-Efficiency MAPbI_3_ Perovskite Solar Cells. Adv. Sci..

[38] Yang Y., Lu H., Feng S., Yang L., Dong H., Wang J., Tian C., Li L., Lu H., Jeong J. (2021). Modulation of perovskite crystallization processes towards highly efficient and stable perovskite solar cells with MXene quantum dot-modified SnO_2_. Energy Environ. Sci..

[39] Yuan R., Cai B., Lv Y., Gao X., Gu J., Fan Z., Liu X., Yang C., Liu M., Zhang W.-H. (2021). Boosted charge extraction of NbO*_x_* -enveloped SnO_2_ nanocrystals enables 24% efficient planar perovskite solar cells. Energy Environ. Sci..

[40] Chen Y., Wang Q., Yao Y., Yang J., Tang W., Qiu W., Wu Y., Peng Q. (2023). Synergistic transition metal ion co-doping and multiple functional additive passivation for realizing 25.30% efficiency perovskite solar cells. Energy Environ. Sci..

[41] Wang D., Guo X., Zhang G., Liu Y., Liu S., Zhang Z., Chai Y., Chen Y., Zhang J., Sun B. (2023). SnO_2_ electron transport layer modified by F/N-doped graphdiyne and in situ XRD and in situ XAFS exploration on its effect on perovskite active layer. Nano Today.

[42] Yan T., Zhang C., Li S., Wu Y., Sun Q., Cui Y., Hao Y. (2023). Multifunctional Aminoglycoside Antibiotics Modified SnO_2_ Enabling High Efficiency and Mechanical Stability Perovskite Solar Cells. Adv. Funct. Mater..

[43] Ji X., Bi L., Fu Q., Li B., Wang J., Jeong S.Y., Feng K., Ma S., Liao Q., Lin F.R. (2023). Target Therapy for Buried Interface Enables Stable Perovskite Solar Cells with 25.05% Efficiency. Adv. Mater..

[44] Zeng X., Zhou T., Leng C., Zang Z., Wang M., Hu W., Tang X., Lu S., Fang L., Zhou M. (2017). Performance improvement of perovskite solar cells by employing a CdSe quantum dot/PCBM composite as an electron transport layer. J. Mater. Chem. A.

[45] Wang Z., Rong X., Wang L., Wang W., Lin H., Li X. (2020). Dual Role of Amino-Functionalized Graphene Quantum Dots in NiO*_x_* Films for Efficient Inverted Flexible Perovskite Solar Cells. ACS Appl. Mater. Interfaces.

[46] Cheng F., He R., Nie S., Zhang C., Yin J., Li J., Zheng N., Wu B. (2021). Perovskite Quantum Dots as Multifunctional Interlayers in Perovskite Solar Cells with Dopant-Free Organic Hole Transporting Layers. J. Am. Chem. Soc..

[47] Lin J., Wang Y., Khaleed A., Syed A.A., He Y., Chan C.C.S., Li Y., Liu K., Li G., Wong K.S. (2023). Dual Surface Modifications of NiO*_x_*/Perovskite Interface for Enhancement of Device Stability. ACS Appl. Mater. Interfaces.

[48] Duan T., You S., Chen M., Yu W., Li Y., Guo P., Berry J.J., Luther J.M., Zhu K., Zhou Y. (2024). Chiral-structured heterointerfaces enable durable perovskite solar cells. Science.

[49] Cao Q., Wang T., Pu X., He X., Xiao M., Chen H., Zhuang L., Wei Q., Loi H.L., Guo P. (2024). Co-Self-Assembled Monolayers Modified NiO*_x_* for Stable Inverted Perovskite Solar Cells. Adv. Mater..

[50] Li Z., Sun X., Zheng X., Li B., Gao D., Zhang S., Wu X., Li S., Gong J., Luther J.M. (2023). Stabilized hole-selective layer for high-performance inverted p-i-n perovskite solar cells. Science.

[51] Kafedjiska I., Levine I., Musiienko A., Maticuic N., Bertmam T., Al-Ashouri A., Kaufmann C.A., Albrecht S., Schlatmann R., Lauermann I. (2023). Advanced Characterization and Optimization of NiO*_x_*: Cu-SAM Hole-Transporting Bi-Layer for 23.4% Efficient Monolithic Cu(In,Ga)Se_2_—Perovskite Tandem Solar Cells. Adv. Funct. Mater..

[52] Nath B., Behera S.K., Kumar J., Hemmerle A., Fontaine P., Ramamurthy P.C., Mahapatra D.R., Hegde G. (2024). Understanding the Heterointerfaces in Perovskite Solar Cells via Hole Selective Layer Surface Functionalization. Adv. Mater..

[53] Zhou Y., Huang X., Zhang J., Zhang L., Wu H., Zhou Y., Wang Y., Wang Y., Fu W., Chen H. (2024). Interfacial Modification of NiO_x_ for Highly Efficient and Stable Inverted Perovskite Solar Cells. Adv. Energy Mater..

[54] Wang X., Jiang J., Liu Z., Li A., Miyasaka T., Wang X. (2024). Zwitterion dual-modification strategy for high-quality NiO_x_ and perovskite films for solar cells. Small.

[55] Cao L., Tong Y., Ke Y., Zhang W., Li T., Kang Z., Wang H., Wang K. (2024). Modification of Nickel Oxide via Self-Assembled Monolayer for Enhanced Performance of Air-Processed FAPbl_3_ Perovskite Solar Cells. ACS Appl. Energy Mater..

[56] Yang Y., Chen R., Wu J., Dai Z., Luo C., Fang Z., Wan S., Chao L., Liu Z., Wang H. (2024). Bilateral Chemical Linking at NiO_x_ Buried Interface Enables Efficient and Stable Inverted Perovskite Solar Cells and Modules. Angew. Chem. Int. Ed..

[57] Zhu H., Xu Z., Zhang Z., Lian S., Wu Y., Zhang D., Zhan H., Wang L., Han L., Qin C. (2024). Improved Hole-Selective Contact Enables Highly Efficient and Stable FAPbBr_3_ Perovskite Solar Cells and Semitransparent Modules. Adv. Mater..

[58] Wang Q., Zhang X., Jin Z., Zhang J., Gao Z., Li Y., Liu S.F. (2017). Energy-Down-Shift CsPbCl_3_: Mn Quantum Dots for Boosting the Efficiency and Stability of Perovskite Solar Cells. ACS Energy Lett..

[59] Liu B., Wang Y., Bian S., Liu Z., Wu Y., Shao L., Zhang Y., Lyu J., Zhang L., Mao J. (2024). Perovskite Solar Cells with Extremely High 24.63% Efficiency through Design of Double Electron Transport Layers and Double Luminescent Converter Layers. Adv. Funct. Mater..

[60] Song P., Hase S., Zhao S., Xu Z., Iso Y., Isobe T. (2022). Feasibility of Emission-Enhanced CsPbCl_3_ Quantum Dots Co-Doped with Mn^2+^ and Er^3+^ as Luminescent Downshifting Layers in Crystalline Silicon Solar Modules. ACS Appl. Nano Mater..

[61] Zhu Y., Dai C., Hao C., Guo H., Yan L. (2022). Purification of nitrogen-doped graphene quantum dots and its application in polymer solar cells. Colloids Surf. A Physicochem. Eng. Asp..

[62] Dey T., Ghorai A., Das S., Ray S.K. (2022). Solvent-engineered performance improvement of graphene quantum dot sensitized solar cells with nitrogen functionalized GQD photosensitizers. Sol. Energy.

[63] Jo I.R., Lee Y.H., Kim H., Ahn K.-S. (2021). Multifunctional nitrogen-doped graphene quantum dots incorporated into mesoporous TiO_2_ films for quantum dot-sensitized solar cells. J. Alloys Compd..

[64] Maity D., Kolay A., Deepa M. (2021). Efficient Charge Separation Enabled by N-Doped Graphene Quantum Dots and PCDTBT for a High-Performance Silicon Nanowire Solar Cell. ACS Appl. Energy Mater..

[65] Bian H., Wang Q., Yang S., Yan C., Wang H., Liang L., Jin Z., Wang G., Liu S. (2019). Nitrogen-doped graphene quantum dots for 80% photoluminescence quantum yield for inorganic γ-CsPbI_3_ perovskite solar cells with efficiency beyond 16%. J. Mater. Chem. A.

[66] Shen D., Lan T., Qiao D., Guo M., Zuo J., Gu S., Zhang H., Li W., Sun X., Li Y. (2024). Tunable Photoluminescent Nitrogen-Doped Graphene Quantum Dots at the Interface for High-Efficiency Perovskite Solar Cells. ACS Appl. Nano Mater..

[67] Tang L., Ji R., Li X., Bai G., Liu C.P., Hao J., Lin J., Jiang H., Teng K.S., Yang Z. (2014). Deep Ultraviolet to Near-Infrared Emission and Photoresponse in Layered N-Doped Graphene Quantum Dots. ACS Nano.

[68] Gao Y., Wu Y., Lu H., Chen C., Liu Y., Bai X., Yang L., Yu W.W., Dai Q., Zhang Y. (2019). CsPbBr3 perovskite nanoparticles as additive for environmentally stable perovskite solar cells with 20.46% efficiency. Nano Energy.

[69] Li M., Yan J., Zhao X., Ma T., Zhang A., Chen S., Shen G., Khalaf G.M.G., Zhang J., Chen C. (2024). Synergistic Enhancement of Efficient Perovskite/Quantum Dot Tandem Solar Cells Based on Transparent Electrode and Band Alignment Engineering. Adv. Energy Mater..

[70] Yang W., Su R., Luo D., Hu Q., Zhang F., Xu Z., Wang Z., Tang J., Lv Z., Yang X. (2020). Surface modification induced by perovskite quantum dots for triple-cation perovskite solar cells. Nano Energy.

[71] Bian H., Bai D., Jin Z., Wang K., Liang L., Wang H., Zhang J., Wang Q., Liu S. (2018). Graded Bandgap CsPbI2+Br1− Perovskite Solar Cells with a Stabilized Efficiency of 14.4%. Joule.

[72] Zheng X., Troughton J., Gasparini N., Lin Y., Wei M., Hou Y., Liu J., Song K., Chen Z., Yang C. (2019). Quantum Dots Supply Bulk- and Surface-Passivation Agents for Efficient and Stable Perovskite Solar Cells. Joule.

[73] Liu D., Guo Y., Yang Y., Liu J., Yin X., Que W. (2022). CuInSe_2_ quantum dots doped MAPbI_3_ films with reduced trap density for perovskite solar cells. J. Alloys Compd..

[74] Xu Y., Ren Y., Cheng S., Zhang L., Niu P., Lyu M., Lu H., Wang M., Zhu J. (2023). A residual strain regulation strategy based on quantum dots for efficient perovskite solar cells. J. Mater. Chem. A.

[75] Chen Y., Chen J., Wang X., Deng P., Shen Y., Wang X. (2025). Fluorinated graphene quantum dots-induced defect passivation of perovskite film toward stable and efficient perovskite solar cell. Int. J. Hydrogen Energy.

[76] Alzahrani Y.A., Alqahtani R.M., Alqarni R.A., Alnakhli J.R., Anezi S.A., Almalki I.S., Yafi G.S., Alenzi S.M., Aljuwayr A., Alessa A.M. (2025). In situ epitaxial quantum dot passivation enables highly efficient and stable perovskite solar cells. Nanomaterials.

[77] Xie L., Vashishtha P., Koh T.M., Harikesh P.C., Jamaludin N.F., Bruno A., Hooper T.J.N., Li J., Ng Y.F., Mhaisalkar S.G. (2020). Realizing Reduced Imperfections via Quantum Dots Interdiffusion in High Efficiency Perovskite Solar Cells. Adv. Mater..

[78] Wang K., Xu Z., Guo Z., Wang H., Qaid S.M.H., Yang K., Zang Z. (2024). Phosphonate Diacid Molecule Induced Crystallization Manipulation and Defect Passivation for High-Performance Inverted MA-Free Perovskite Solar Cells. Adv. Energy Mater..

[79] Gao F., Zheng Q., Zhang Y. (2019). Stability Improvement of Perovskite Solar Cells for Application of CuInS_2_ Quantum Dot-Modified TiO_2_ Nanoarrays. ACS Omega.

[80] Hoffman J.B., Zaiats G., Wappes I., Kamat P.V. (2017). CsPbBr_3_ Solar Cells: Controlled Film Growth through Layer-by-Layer Quantum Dot Deposition. Chem. Mater..

[81] Ghosh D., Chaudhary D.K., Ali M.Y., Chauhan K.K., Prodhan S., Bhattacharya S., Ghosh B., Datta P.K., Ray S.C., Bhattacharyya S. (2019). All-inorganic quantum dot assisted enhanced charge extraction across the interfaces of bulk organo-halide perovskites for efficient and stable pin-hole free perovskite solar cells. Chem. Sci..

[82] Jiang J., Li R., Liu D., Xie H., Zeng Q., Li Y. (2023). Efficient and Stable CsPbI_2_ Br Inorganic Perovskite Solar Cell Co-Modified with Ionic Liquids and Quantum Dots. ACS Appl. Energy Mater..

[83] Que M., Dai Z., Yang H., Zhu H., Zong Y., Que W., Padture N.P., Zhou Y., Chen O. (2019). Quantum-Dot-Induced Cesium-Rich Surface Imparts Enhanced Stability to Formamidinium Lead Iodide Perovskite Solar Cells. ACS Energy Lett..

[84] Zhang Y., Gu M., Li N., Xu Y., Ling X., Wang Y., Zhou S., Li F., Yang F., Ji K. (2018). Realizing solution-processed monolithic PbS QDs/perovskite tandem solar cells with high UV stability. J. Mater. Chem. A.

[85] Dong C., Yan S., Liu D., Zhu Y., Chen C., Tang J. (2025). Principle and progress of interconnection layers in monolithic perovskite-based tandem photovoltaics. Adv. Energy Mater..

[86] Becker-Koch D., Albaladejo-Siguan M., Lami V., Paulus F., Xiang H., Chen Z., Vaynzof Y. (2020). Ligand dependent oxidation dictates the performance evolution of high efficiency PbS quantum dot solar cells. Sustain. Energy Fuels.

[87] Er-Raji O., Lange S., Hartwig C.E., Prasetio A., Bivour M., Hermle M., Turek M., De Wolf S., Glunz S.W., Borchert J. (2025). Tuning self-assembly of hole-selective monolayers for reproducible perovskite/silicon tandem solar cells. Small Methods.

[88] Fang Z., Ding L., Yang Y., Gu X., Li H., Chen H., Yin Y., Wang W., Wu X., Rao Z. (2026). Flexible perovskite/silicon tandem solar cell with a dual-buffer layer. Nature.

[89] Lin R., Gao H., Lou J., Xu J., Yin M., Wu P., Liu C., Guo Y., Wang E., Yang S. (2025). All-perovskite tandem solar cells with dipolar passivation. Nature.

